# The FLASH effect—an evaluation of preclinical studies of ultra-high dose rate radiotherapy

**DOI:** 10.3389/fonc.2024.1340190

**Published:** 2024-04-22

**Authors:** Josie May McGarrigle, Kenneth Richard Long, Yolanda Prezado

**Affiliations:** ^1^ Department of Physics, Imperial College London, London, United Kingdom; ^2^ Science and Technology Facilities Council (STFC), Rutherford Appleton Laboratory, Oxford, United Kingdom; ^3^ Institut Curie, Universite Paris-Saclay, Centre national de la recherche scientifique (CNRS) UMR3347, Inserm U1021, Signalisation radiobiologie et cancer, Orsay, France; ^4^ Universite Paris-Saclay, Centre national de la recherche scientifique (CNRS) UMR3347, Inserm U1021, Signalisation radiobiologie et cancer, Orsay, France

**Keywords:** ultra-high dose rate irradiation, ultra-high dose rate, FLASH-RT, normal-tissue sparing, FLASH, radiotherapy, therapeutic index, radiation - adverse effects

## Abstract

FLASH radiotherapy (FLASH-RT) is a novel radiotherapy approach based on the use of ultra-high dose radiation to treat malignant cells. Although tumours can be reduced or eradicated using radiotherapy, toxicities induced by radiation can compromise healthy tissues. The FLASH effect is the observation that treatment delivered at an ultra-high dose rate is able to reduce adverse toxicities present at conventional dose rates. While this novel technique may provide a turning point for clinical practice, the exact mechanisms underlying the causes or influences of the FLASH effect are not fully understood. The study presented here uses data collected from 41 experimental investigations (published before March 2024) of the FLASH effect. Searchable databases were constructed to contain the outcomes of the various experiments in addition to values of beam parameters that may have a bearing on the FLASH effect. An in-depth review of the impact of the key beam parameters on the results of the experiments was carried out. Correlations between parameter values and experimental outcomes were studied. Pulse Dose Rate had positive correlations with almost all end points, suggesting viability of FLASH-RT as a new modality of radiotherapy. The collective results of this systematic review study suggest that beam parameter qualities from both FLASH and conventional radiotherapy can be valuable for tissue sparing and effective tumour treatment.

## 1 Introduction

Cancer is an accumulation of abnormal, malignant cells which reproduce uncontrollably. In radiotherapy, a beam of ionising radiation is used to control or eliminate these malignant cells. The beam characteristics are optimised to maximise the impact on the cancer while minimising damage to healthy tissues. Radiotherapy is commonly delivered over several days in fractions of ∼ 2Gy, each fraction being delivered at a rate of $\underset{∼}{<}$ 10Gy/min. There is now a body of evidence that suggests that the delivery of the therapeutic dose at very high rates in FLASH Radiotherapy (FLASH-RT) yields a degree of tumour control equivalent to conventional radiotherapy while significantly reducing the adverse toxicities (1).

Studies of FLASH-RT are usually categorised using two parameters: tumour response and normal-tissue response. Previous studies have found that the normal-tissue response to FLASH-RT includes less clustered DNA damage sites, fewer dicentric chromosomes and a smaller fraction of G2 cells than are present in the response to conventional radiotherapy (2). While there are hypotheses on the processes that may give rise to the FLASH effect (3), its origin is unknown and the current understanding of the factors that influence the FLASH effect is limited.

In conjunction with dose rates, contradictory biological results from experiments performed with different beams resulted in the hypothesis that the beam parameters influence the appearance of the FLASH effect. This review explores the dependency of the FLASH effect on key irradiation parameters. The biological response induced by FLASH radiotherapy (FLASH-RT) is discussed, providing insights into its potential clinical applications.

## 2 Materials and methods

The primary focus of this study was the search for patterns in the data from experiments in which the FLASH effect had been investigated. Searchable databases were created in which the outcomes of FLASH radiotherapy experiments were stored alongside the parameters that determined the irradiation conditions. The database was then used to search for patterns in the results to determine which parameters most influence the normal-tissue and tumour responses to FLASH-RT.

A critical investigation of 41 experiments was performed to derive quantitative measures of the eradication of malignant cells and the creation of lasting side effects (e.g. skin damage and other toxicities). Examining each study in isolation allowed parameters to be identified that might be correlated to the measures of tumour control and normal-tissue sparing. By combining the data from all experiments, the analysis presented here seeks to overcome the statistical limitations of the individual studies, each of which is insufficient on its own to establish a clear connection between a particular parameter and the onset of the FLASH effect.

The crux of FLASH-RT is the increase of the therapeutic window through the delivery of the therapeutic dose at ultra-high dose rate. It has therefore been presupposed that the parameter that would be most directly correlated to the FLASH effect would be the dose rate. A number of preliminary studies such as the 2017 experiment conducted by Montay-Gruel et al. (4), which showed that higher dose rates were correlated with memory sparing, suggested this correlation. In order to elucidate the conditions in which a FLASH effect can be observed, it is imperative to expand these data sets and look for an overarching trend that is present in all, or many, studies.

### 2.1 Search criteria

The parameters identified as potentially correlated with the FLASH effect and considered in this study are:

- Mean Dose Rate (Gy/s)–the average dose rate across the duration of the irradiation;
- Pulse Dose Rate (Gy/s)–the dose rate delivered by each individual pulse, each pulse being composed of a number of bunches from the accelerator;
- Pulse Dose (Gy)–the dose in each pulse;
- Total Dose (Gy)–the total administered dose;
- Pulse Width (*µ*s)–the temporal duration of each pulse;
- Total Duration (s)–the total time taken to administer the full dose;
- Repetition Frequency (Hz)–the frequency at which pulses are delivered; and
- Number of Pulses–the number of pulses delivered.

Data from papers published before March 2024 were considered for inclusion in the present study. The study evaluated each paper using the categories of: population, intervention, comparison, outcome (PICO) as a search strategy tool. The categories used to collect data for this systematic review study are listed in **Table 1**.

**Table 1:** Population, Intervention, Comparison, Outcome (PICO) search strategy used to select relevant experiments.

Population	Intervention
*In-VITRO* cells/Small animal *In-VIVO* models	FLASH Radiotherapy
Comparison	Outcome
Control group/Pre-radiation	Biological Response Described/Quantified

A more extensive description of each study and an explanation of the coherence to these qualities is listed in the **Supplementary Materials**. The specific tumour, tissue and cell types are listed in **Tables 2**, **3**; **Table 2** displays the normal-tissue experiments and **Table 3** displays the tumour experiments.

**Table 2:** A table of normal-tissue types in relevant experiments.

Environment	Species	Cell Line	Irradiation Area	Reference
*in-vivo*	Mouse	N/A	Brain	(4–11)
*in-vivo*	Mouse	N/A	Abdomen	(12–15)
*in-vivo*	Mouse	N/A	Skin	(16–21)
*in-vivo*	Mouse	N/A	Lung	(14, 22–24)
*in-vivo*	Rat	N/A	Skin	(25)
*in-vivo*	Mouse	N/A	Thorax	(14, 23)
*in-vivo*	Mouse	N/A	Heart	(15)
*in-vivo*	Mouse	N/A	Spleen	(15)
*in-vivo*	Cat	N/A	Skin	(26)
*in-vivo*	Minipig	N/A	Skin	(26)
*in-vivo*	Rat	N/A	Brain	(27)
*in-vivo*	Mouse	Human M106 T-cells	Blood	(28)
*in-vivo*	Mouse	N/A	Pelvis	(29)
*in-vitro*	Zebrafish	N/A	Embryo	(30, 31)
*in-vitro*	Human	IMR-90	N/A	(32)
*in-vitro*	Human	N/A	Blood	(33)
*in-vitro*	Human	Epithelium Cell Line 184A1	N/A	(34)
*in-vitro*	Human	HeLa Cells	N/A	(35)

**Table 3:** A table of tumour types in relevant experiments.

Environment	Species	Cell Line	Tumour Type	Reference
*in-vivo*	Cat	T2/T3N0M0 Squamous Cell Carcinoma of the Nasal Planum	Skin	(26)
*in-vivo*	Rat	NS1 Rat Glioma	Brain	(27, 36)
*in-vivo*	Mouse	C57BL/6J Mouse GL261/Human U-87 MG	Brain	(37)
*in-vivo*	Mouse	B16–F10 Melanoma Cells	Skin	(38)
		MOC1/MOC2 (Mouse Oral Squamous Cell Carcinoma)		(19)
		B-16 Flank Tumour		(29)
*in-vivo*	Mouse	ID8 Ovarian Cancer	Abdomen	(12, 39)
*in-vivo*	Mouse	FaDu	Head and Neck	(40)
		HEp-2 Xenografts		(23)
*in-vivo*	Mouse	C3H Mouse Mammary Carcinoma	Breast	(20)
		EMT6 Mouse Breast Cancer		(14)
		HBCx-12A Ductal Carcinoma		(23)
*in-vivo*	Mouse	LLC Lewis Lung Carcinoma	Lung	(41, 42)
		TC-1 Cells (C57BL/6J Mouse Lung Carcinoma)		(23)
*in-vivo*	Mouse	Human M106 T-cells	Blood	(28)
*in-vitro*	Human	DU145	Prostate	(43)
*in-vitro*	Human	FaDu	Head and Neck	(34)
*in-vitro*	Mouse	KPC & Panc02	Pancreas	(15)
		Pancreatic Flank Tumours MH641905		(13)
*in-vitro*	Human	MCF-7 Breast Adenocarcinoma Cells	Breast	(44)

### 2.2 Data analysis

A set of metrics was developed to allow a quantitative comparison of the varying endpoints that have been reported. Experiments which had determined the degree to which irradiation induced moist desquamation, impaired cognitive function, induced changes in stool level, tumour size, survival fractions, and/or caused fibrosis were included in this study. Unfortunately, each of these endpoints was examined in not more than one or two of the experiments. Scores were manually assigned to these endpoints to reflect the degree to which tumour control had been generated and normal-tissue sparing observed.

Some experiments did not record *both* tumour control and normal-tissue sparing, meaning that some scores had to be based on tumour control or normal-tissue sparing alone. Consequently, tumour response and normal-tissue sparing were individually scored. Scoring in this way would also account for the fact that larger doses and dose-delivery periods would typically result in more effective tumour control at the expense of increased normal-tissue toxicity. The criteria against which the scores were assigned in the two categories “Tumour Control” and “Normal-tissue Sparing” are defined in **Table 4**. The end-point in each experiment was evaluated, converting the qualitative result into a number between 1 and 5 (fractional scores were awarded if the result fell between two categories). The overall score (to evaluate the degree to which FLASH-RT is effective as a radiotherapy treatment modality) was created by averaging the tumour-control and normal-tissue-sparing scores from experiments that evaluated *both* tumour control and normal-tissue sparing.

**Table 4:** Scoring system used to quantify the experimental biological responses.

Tumour Control Score (TCS)			Normal-tissue Sparing Score (NTSS)		
Score	Metric	Example	Score	Metric	Example
1	No tumour control	e.g. tumour same size, no shrinkage	1	No radio-protection	e.g. no normal-tissue sparing, high damage
2	Small amount of tumour control	e.g. slight shrinkage/little short term control	2	Low level of radioprotection	e.g. little normal-tissue sparing, noticeable damage
3	Moderate tumour control	e.g. some shrinkage or short term control	3	Moderate radio-protection	e.g. some normal-tissue sparing ob- served, little damage
4	Fair tumour control	e.g. short term control, potential long term but recurrence	4	Fair radio-protection	e.g. normal-tissue sparing observed, minimal damage
5	Complete tumour control	e.g. short/long term control, no recurrence	5	Great radio-protection	e.g. complete normal-tissue preservation, no damage

In this paper, the quantitative tumour-control score is labelled “Tumour Control Score” or TCS, the normal-tissue-sparing score is labelled “Normal-tissue Sparing Score” or NTSS, and the overall score was labelled “Therapeutic Index Score” or TIS. The scores were used to evaluate the hypothesis that FLASH irradiation yields reduced normal-tissue sparing but maintains tumour control for each beam parameter identified in section 2.1. The database, including the calculated Therapeutic Index Score (with explanations of each score), is given in the **Supplementary Materials**.

The assessment of small animal survival in this review study allows for a comprehensive examination of the late-stage impacts of FLASH-RT, emphasising a focus beyond the commonly explored acute effects. This approach defines the two modes of survival statistics: “Survival Score” (S*_M_*) and “Increased Lifespan” (ILS). S*_M_* refers to the percentage of survivors (S) still alive at different time points (M) after treatment and is defined by Equation 1:


(1) $${\text{S}}_{M}=\frac{\text{Animals alive M months post treatment}}{\text{Animals irradiated}}\;\times 100\;.$$

In this study, survivors were recorded at *M* = 1, 2 and 3 months for each experiment. ILS is defined in Equation 2 as the ratio of treated to untreated median survival time (MST):


(2) $$\text{ILS}=\frac{\left({\text{FLASH}}_{\text{MST}}-{\text{control}}_{\text{MST}}\right)}{{\text{control}}_{\text{MST}}}\;\times 100\;;$$

where FLASH_MST_ is the “median survival time”, the time at which the number of survivors drops below 50% post FLASH-RT and Control_MST_ is the median survival time of the untreated group.

The bivariate Pearson’s correlation coefficient, *r*, was calculated to quantify the degree of any correlation between the chosen parameter and the FLASH response. Pearson’s correlation coefficient is given by Equation 3:


(3) $$r=\frac{\sum_{i=1}^{n}(x_{i}-\bar{x})\left(y_{i}-\bar{y}\right)}{\sqrt{\sum_{i=1}^{n}{\left(x_{i}-\bar{x}\right)}^{2}}\sqrt{\sum_{i=1}^{n}{\left(y_{i}-\bar{y}\right)}^{2}}}\;;$$

where *n* is the number of measurements in the sample, the *x_i_* are the values of the beam parameters, the *y_i_* are the associated quantitative outcomes, and the mean of the *x_i_* and *y_i_* are $\bar{x}$ and $\bar{y}$ respectively. In the following, |*r* | *>* 0.5 is categorised as a “strong correlation”, *r* in the range 0.3<|*r*| ≤ 0.5 is categorised as a moderate correlation, and |*r*| ≤ 0.3 is categorised as a weak correlation.

A confidence-level analysis to establish the degree to which the null hypothesis, that the outcome is uncorrelated with the beam parameter, can be rejected was carried out by calculating the test statistic, *t*, given by Equation 4:


(4) $$t=r{\left(\frac{n-2}{1-r^{2}}\right)}^{\frac{1}{2}}\;.$$

where r is the Pearson correlation coefficient and *n* is the size of the sample.

For the null hypothesis, *r* = 0 and *t* follows the Student’s *t* distribution with *n*−2 degrees of freedom. The confidence level, or “*p*-value”, was evaluated as the probability that a value with magnitude ≤ |*t*| would occur by chance. For this review study, statistical significance is characterised as a p-value of less than 0.05.

As a cross-check, the standard deviation of the data points from the value expected based on the null hypothesis (*σ_null_*), that the points and beam parameter are uncorrelated and the alternative hypothesis (*σ*), that the points are correlated with the beam parameter, were calculated. The standard deviations are defined by Equations 5 and 6 respectively:


(5) $$\sigma_{null}=\sqrt{\frac{\sum {\left(y_{i}-\bar{y}\right)}^{2}}{n-1}}\;;$$

(6) $$\sigma =\sqrt{\frac{\sum {\left(y_{i}-y_{est}\right)}^{2}}{n-2}}\;;$$

where *y*_est_ is the estimate of the end-point score obtained from the line of best fit, and *n* is the number of data points. If *σ < σ_null_*, it is more likely that the end point is correlated with the beam parameter, while, if *σ_null_* < *σ*, it is more likely that the data and beam parameter are uncorrelated.

To visually examine the trends observed in the data, each scored endpoint was evaluated and plotted as a function of the most significant beam parameter of the dataset. A straight line fit was performed on each graph to allow the correlation to be visualised. The straight-line fit was used to determine the 95% confidence interval CI and the 95% prediction interval PI calculated using Equations 7 and 8 respectively.:


(7) $$\text{CI}=\hat{y}\pm t_{\text{crit}}\cdot \sigma \sqrt{\frac{1}{n}+\frac{{\left(x-\bar{x}\right)}^{2}}{{\left(\hat{x}-\bar{x}\right)}^{2}}}\;;\text{and}$$

(8) $$\text{PI}=\hat{y}\pm t_{\text{crit}}\cdot \sigma \sqrt{1+\frac{1}{n}+\frac{{\left(x-\bar{x}\right)}^{2}}{{\left(\hat{x}-\bar{x}\right)}^{2}}};$$

where $\hat{y}$ is the end point values, *t*_crit_ is the statistic of interval confidence also known as the critical value of the t distribution [ (45)], *σ* is the squared deviation of the end point values, *x* is an array of evenly spaced x values for the range of each beam parameter, $\bar{x}$ is the mean of the beam parameters. The expected distribution of the data is not known; therefore, this approach is primarily employed to investigate correlated trends and illustrate their spread, rather than presuming linearity or normal distribution of the data.

## 3 Results

The experiments included in this study were carried out at 17 different beam lines and the data was collected using a wide variety of beam parameters. The value of some of the beam parameters varied significantly between experiments so the values of some of the beam parameters spanned a very wide range. It was therefore convenient to study the correlation of the various scored end-points for each beam parameter, *x_b_*, as well as for its logarithm, log_10_(*x_b_*), in order to compress the range of values and spread them out more evenly along the axis. The analysis of the data as a function of log_10_ *x_b_* was more compelling for this reason (see **Supplementary Materials**) and the analysis presented in this study will use log_10_ *x_b_*.

### 3.1 Therapeutic Index Scores- TIS

**Figure 1:**
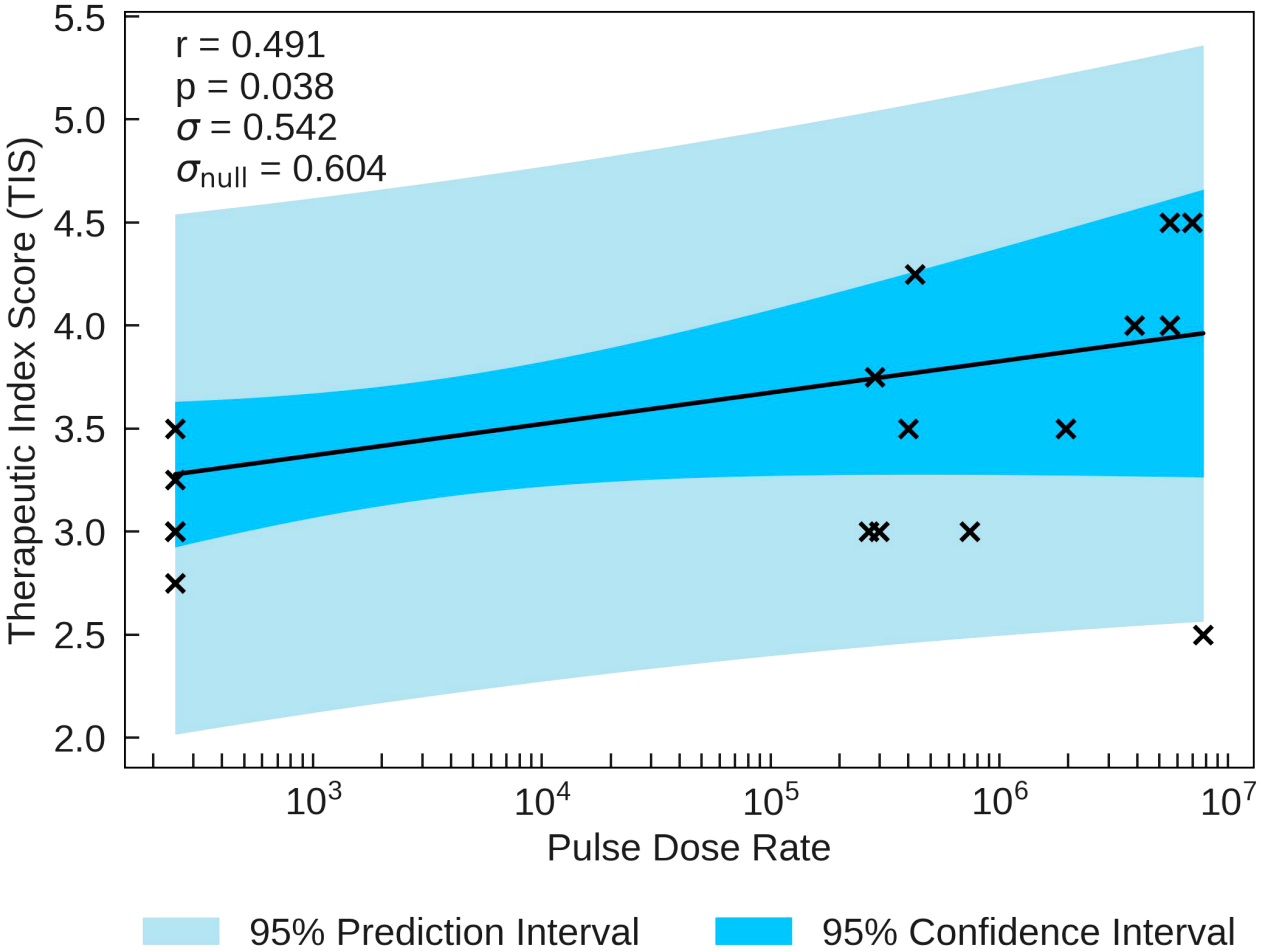
TIS plotted against the most significant and strongest beam parameter, Pulse Dose Rate. A positive correlation between the parameters suggests that an increase in dose rate will increase the chance of observing a higher therapeutic index, as predicted in section 2.

The statistical analysis of the correlation of Therapeutic Index Scores (TIS) with the beam parameters is presented in **Table 5**. TIS is most significantly correlated with Pulse Dose Rate (*r* = 0.491). TIS is plotted as a function of Pulse Dose Rate in **Figure 1**. The statistical significance for this correlation (*p* = 0.038) combined with the value of *σ* being smaller than *σ*_null_ validates the strength of the correlation, providing evidence of a potential FLASH effect. Pulse Dose is also statistically significant, with a p-value of *p* = 0.046 and moderate positive correlation of *r* = 0.476. *σ* is also much smaller than *σ*_null_ for this correlation.

**Table 5:** Statistical analysis for TIS data.

Beam Parameter	*r*	*p*	*σ*	*σ*_null_
Mean Dose Rate (Gy/s)	0.228	0.203	0.688	0.696
Pulse Dose (Gy)	0.476	0.046*	0.547	0.604
Pulse Dose Rate (Gy/s)	0.491	0.038*	0.542	0.604
Repetition Frequency (Hz)	-0.079	0.755	0.620	0.604
Pulse Width (µs)	-0.345	0.161	0.584	0.604
Number of Pulses	-0.448	0.062	0.556	0.604
Total Dose (Gy)	0.026	0.875	0.719	0.709
Total Duration (s)	-0.215	0.229	0.690	0.696

r - Pearson Correlation Coefficient, p - p-value (significance), σ-residual standard deviation, σ_null_ - residual standard deviation for a 0 correlation. Statistically significant correlations are identifiable by an asterisk.

### 3.2 Tumour Control Scores- TCS

The correlations of TCS with the various beam parameters are illustrated in **Table 6**. The most significant correlation

**Table 6:** Statistical analysis for TCS data.

Beam Parameter	*r*	*p*	*σ*	*σ*_null_
Mean Dose Rate (Gy/s)	0.105	0.437	1.175	1.171
Pulse Dose Rate (Gy/s)	0.270	0.123	1.065	1.089
Pulse Width (µs)	-0.141	0.433	1.104	1.098
Pulse Dose (Gy)	0.300	0.096	1.073	1.106
Repetition Frequency (Hz)	0.272	0.132	1.082	1.106
Number of Pulses	-0.075	0.685	1.121	1.106
Total Dose (Gy)	0.280	0.021*	1.127	1.165
Total Duration (s)	0.006	0.966	1.181	1.171

r - Pearson Correlation Coefficient, p - p-value (significance), σ-residual standard deviation, σ_null_ - residual standard deviation for a 0 correlation. Statistically significant correlations are identifiable by an asterisk.

between TCS and the beam parameters on a logarithmic scale is with Total Dose (*r* = 0.280, *p* = 0.021). The *σ* value being smaller than *σ*_null_ along with the statistical significance indicate that the correlation, while not strong, gives a reasonable description of the data. TCS is plotted as a function of Total Duration in **Figure 2**. This correlation suggests that the tumour response is as expected in classical radiobiology, supported by the non-significant moderately positive correlation between Pulse Dose and TCS (*r* = 0.300). Conversely, Pulse Dose Rate and TCS also held a non-significant weak positive correlation (*r* = 0.270). With a stronger, statistically significant correlation, this would suggest that new radiotherapy models such as FLASH has the potential for effective tumour treatment.

**Figure 2:**
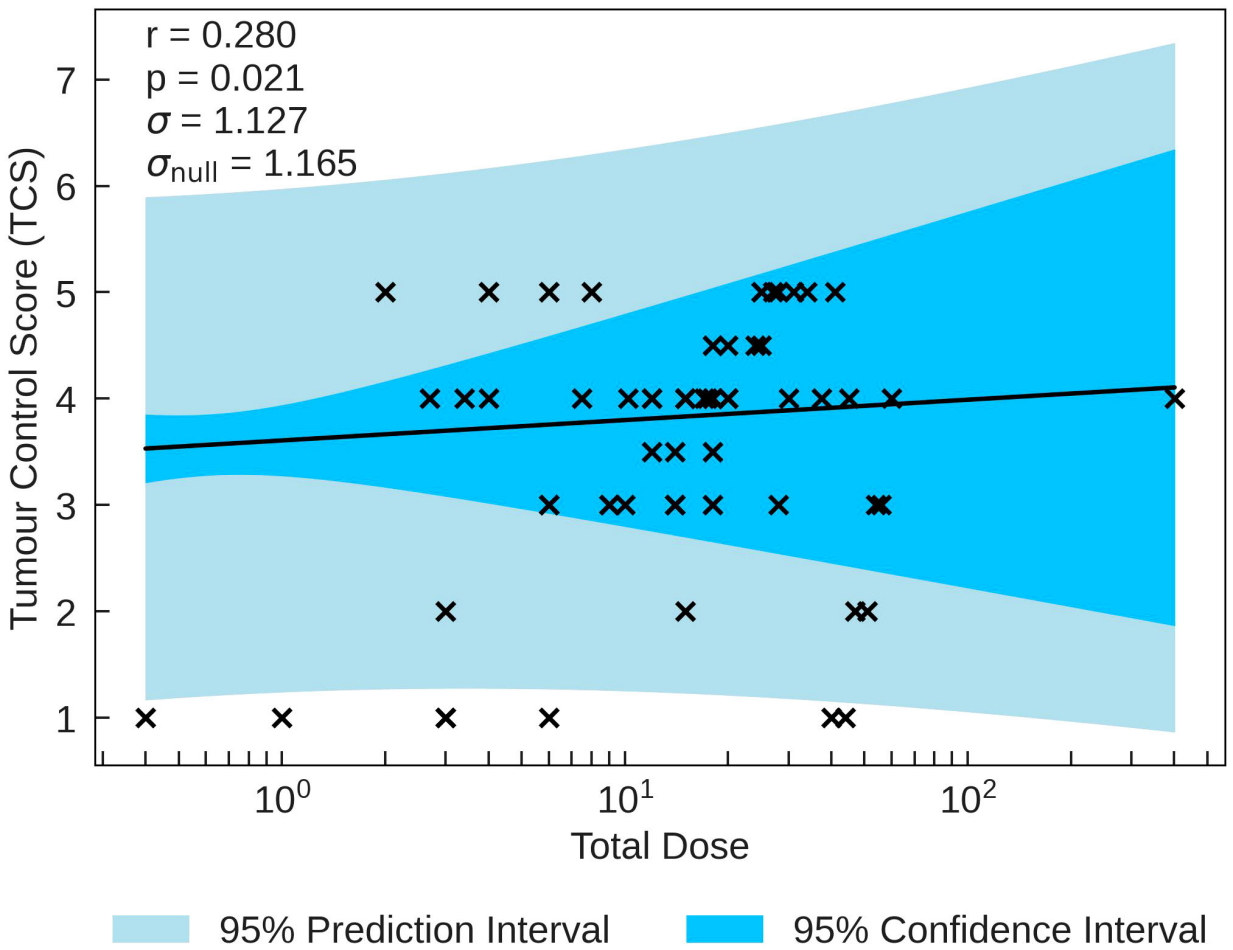
TCS plotted against the most significant and strongest beam parameter, Total Dose. A positive correlation between the parameters suggests that an increase in irradiation dose increases tumour control.

### 3.3 Normal-tissue Sparing Scores- NTSS

**Figure 3:**
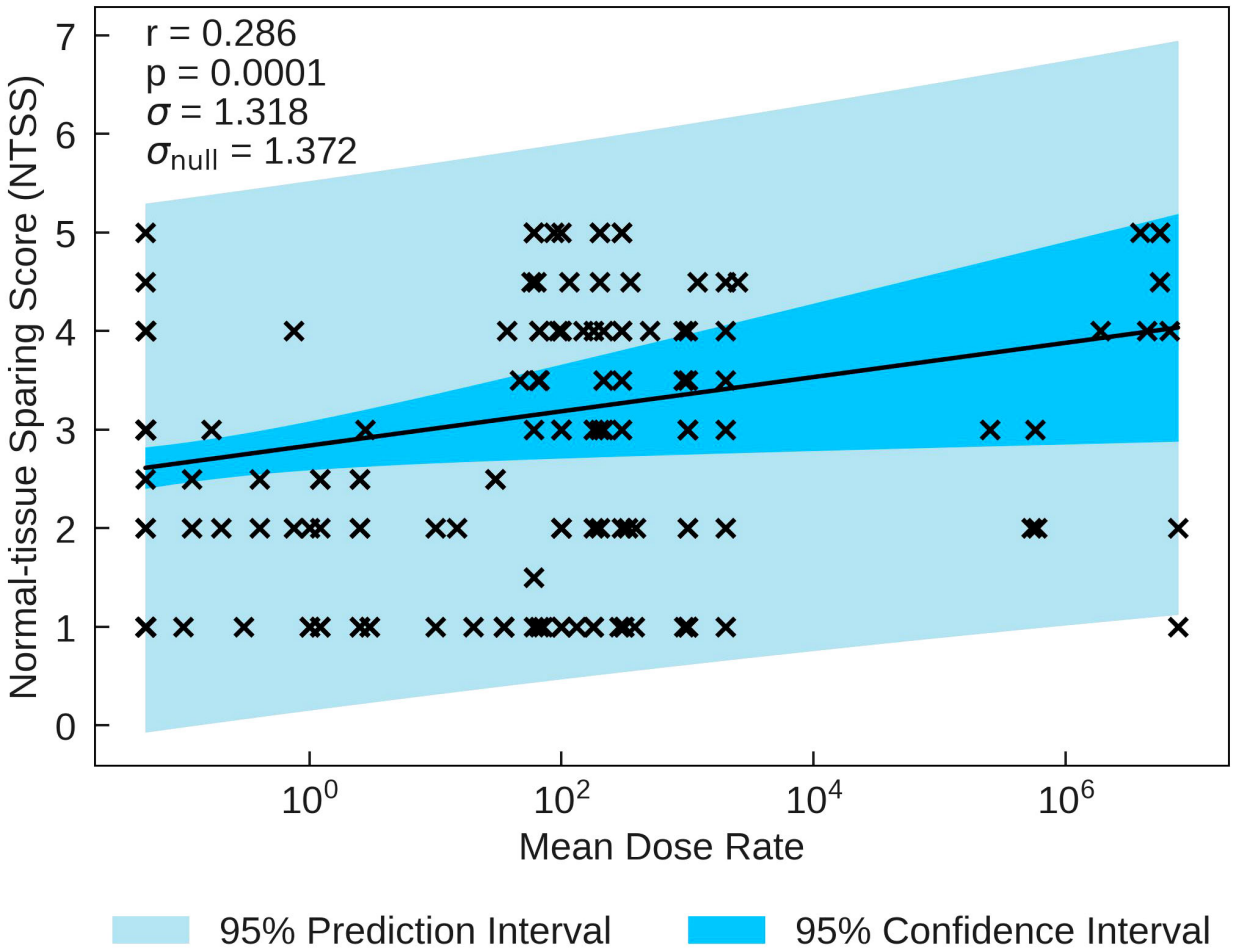
NTSS plotted against the most statistically significant beam parameter, Mean Dose Rate. A positive correlation between the parameters suggests that a decrease in pulse rate may increase the chance of observing a sparing effect in normal tissue.

The statistics characterising the correlations of NTSS with the various parameters are summarised in **Table 7**. NTSS has the most statistically significant correlation with Mean Dose Rate (*r* = 0.286, *p* = 0.0001). This correlation, plotted in **Figure 3**, suggests that an increase in the dose rate may increase the chance of observing sparing in normal-tissue, showing evidence of a FLASH effect, supported by NTSS statistically significant correlations with Pulse Dose Rate (*r* = 0.226, *p* = 0.019) and Total Duration (*r* = −0.222, *p* = 0.004). This is challenged by the two other statistically significant NTSS correlations; Number of Pulses (*r* = −0.410, *p* = 0.001) and Pulse Dose (*r* = 0.367, *p* = 0.001), suggesting attributes of conventional therapy are sufficient for tissue sparing.

**Table 7:** Statistical analysis for Normal-tissue Sparing scored data.

Beam Parameter	*r*	*p*	*σ*	*σ*_null_
Mean Dose Rate (Gy/s)	0.286	0.0001*	1.318	1.372
Pulse Dose Rate (Gy/s)	0.226	0.019*	1.317	1.346
Pulse Width (µs)	0.163	0.130	1.350	1.361
Pulse Dose (Gy)	0.367	0.001*	1.259	1.344
Repetition Frequency (Hz)	-0.111	0.332	1.345	1.344
Number of Pulses	-0.410	0.001*	1.265	1.376
Total Dose (Gy)	-0.0001	0.999	1.392	1.388
Total Duration (s)	-0.222	0.004*	1.359	1.389

r - Pearson Correlation Coefficient, p - p-value (significance), σ - residual standard deviation, σ_null_ - residual standard deviation for a 0 correlation. Statistically significant correlations are identifiable by an asterisk.

### 3.4 Increased Lifespan Score- ILS

**Figure 4:**
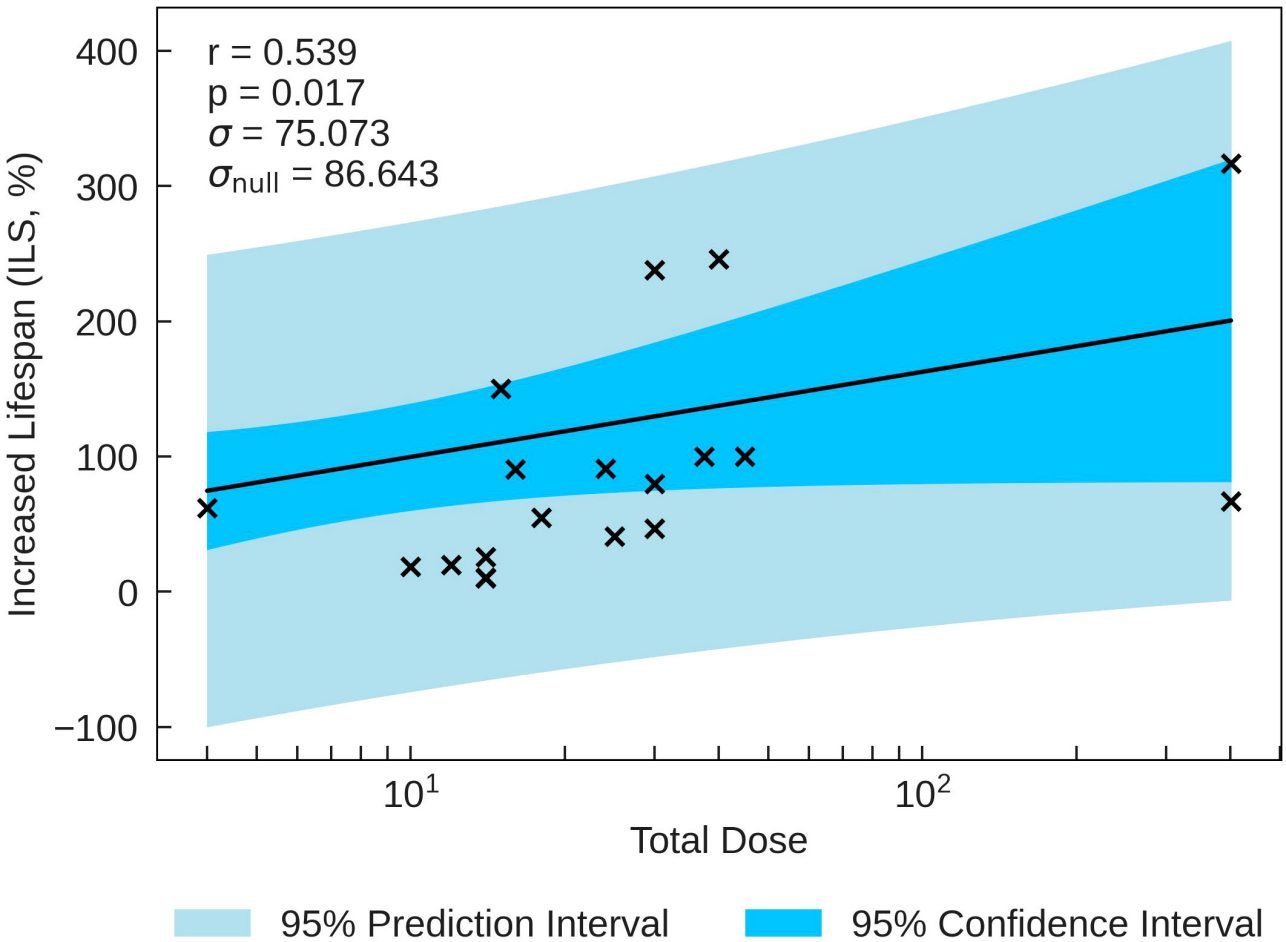
ILS plotted against the most significant and strongest beam parameter, Total Dose. A strong positive correlation between the parameters implies that an increase in dose can increase the lifespan of small animals.

The statistics characterising the ILS correlation with the various parameters are summarised in **Table 8**. The most statistically significant ILS correlation is with Total Dose (*r* = 0.539, *p* = 0.017), closely followed by Total Duration (*r* = 0.536, *p* = 0.022. These correlations are plotted in **Figure 4**. The *σ* values for these correlations are less than *σ*_null_. In conjunction with other correlations presented in **Table 8**, the results suggest that higher doses and dose times yield greater lifespan increases. This also suggests that ILS is dominated by tumour control.

**Table 8:** Statistical analysis for Increased Lifespan.

Beam Parameter	*r*	*p*	*σ*	*σ*_null_
Mean Dose Rate (Gy/s)	-0.418	0.084	81.731	87.276
Pulse Dose Rate (Gy/s)	-0.318	0.248	71.369	72.536
Pulse Width (µs)	0.290	0.295	72.043	72.536
Pulse Dose (Gy)	-0.013	0.962	75.267	72.536
Repetition Frequency (Hz)	0.052	0.853	75.170	72.536
Number of Pulses	0.379	0.164	69.667	72.536
Total Dose (Gy)	0.539	0.017*	75.073	86.643
Total Duration (s)	0.536	0.022*	75.964	87.276

r - Pearson Correlation Coefficient, p - p-value (significance), σ - residual standard deviation, σ_null_ - residual standard deviation for a 0 correlation. Statistically significant correlations are identifiable by an asterisk.

### 3.5 Survival Score- SS

**Figure 5:**
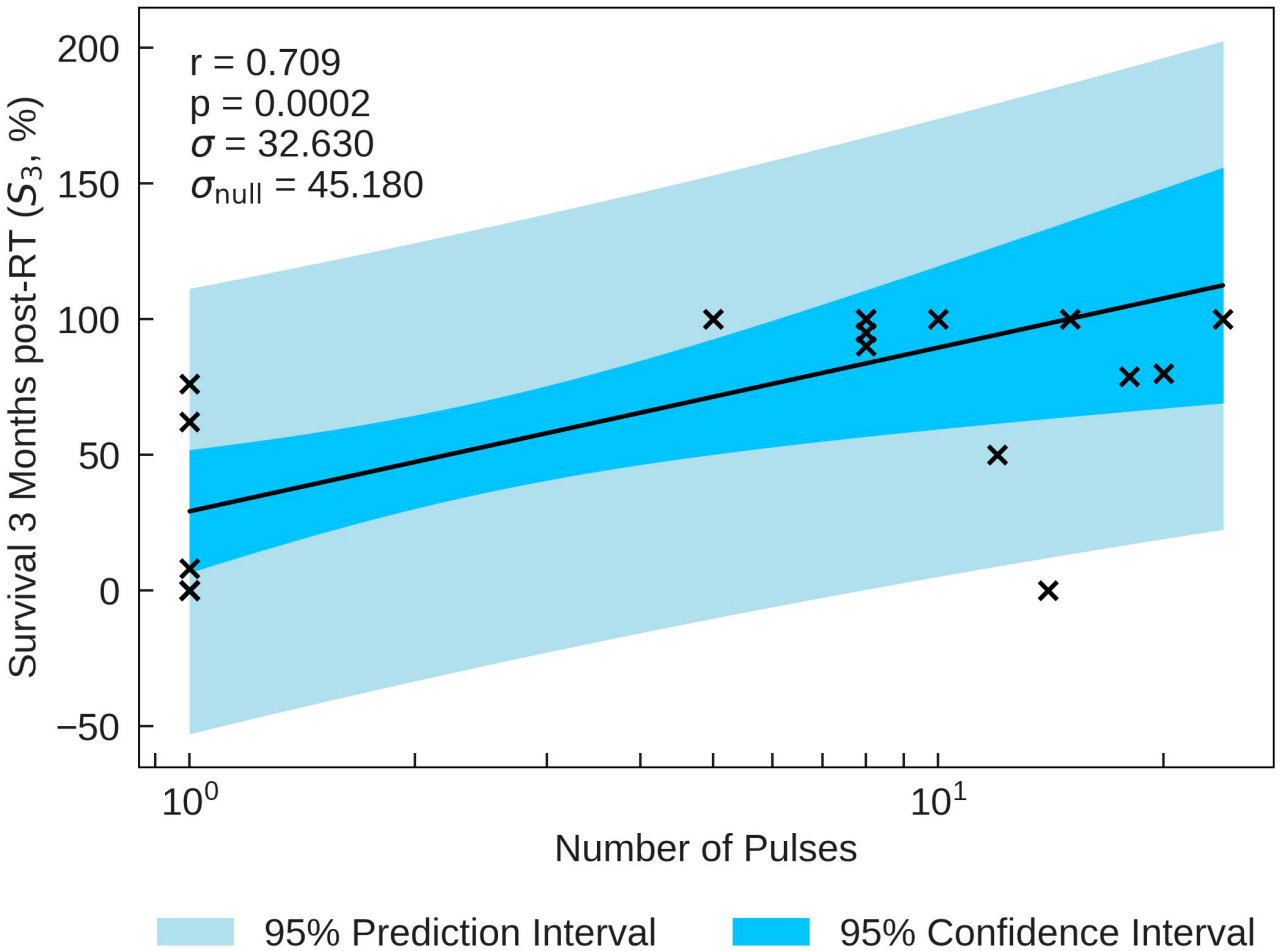
S_3_ percentage plotted against the most significant and strongest beam parameter, Number of Pulses. A strong positive correlation between the parameters implies that an increase in the Number of Pulses can increase the survival time of small animals.

The statistics characterising the correlation of the Survival Score (SS) at 3 months post FLASH-RT with the various parameters are summarised in **Table 9**. SS is most significantly correlated with Number of Pulses (*r* = 0.709, *p* = 0.0002), plotted in **Figure 5**. The correlations with Pulse Dose and Mean Dose Rate are also statistically significant (*r*= −0.600, *p*=0.003 and *r*=−0.442, *p*=0.035 respectively), suggesting that there is a negative relation between the survival of small animals and dose rates at 3 months.

**Table 9:** Statistical analysis for survivors at 3 months.

Beam Parameter	*r*	*p*	*σ*	*σ*_null_
Mean Dose Rate (Gy/s)	-0.442	0.035*	41.731	45.475
Pulse Dose Rate (Gy/s)	0.114	0.621	47.127	46.237
Pulse Width (µs)	-0.254	0.266	45.877	46.237
Pulse Dose (Gy)	-0.600	0.003*	37.030	45.180
Repetition Frequency (Hz)	0.294	0.195	45.338	46.237
Number of Pulses	0.709	0.0002*	32.630	45.180
Total Dose (Gy)	-0.0721	0.738	45.427	44.545
Total Duration (s)	0.434	0.039*	41.922	45.475

r - Pearson Correlation Coefficient, p - p-value (significance), σ - residual standard deviation, σ_null_ - residual standard deviation for a 0 correlation. Statistically significant correlations are identifiable by an asterisk.

### 3.6 Discussion

The FLASH effect is an intriguing radiobiological effect, holding promise to revolutionise radiotherapy. However, the exact conditions to activate the FLASH effect are still unclear. For that purpose, a retrospective evaluation has been carried out to study the FLASH dependence on beam parameters.

This review study highlighted that TIS, TCS, and NTSS showed low correlations with most of the beam parameters. A possible reason for the observed low correlation was explored. This was the presence of a dose-rate threshold for the onset of the FLASH effect. For example, Boreham et al. (46) first explored the dose rate thresholds in early 2000. Their study demonstrated that decreasing the dose rate from 702mGy/min to 2.9 mGy/min had a negligible impact on the induction of lymphocyte apoptosis. However, once the dose rate surpassed 1.5 mGy/min, the rate of lymphocyte apoptosis was significantly reduced. This effect was thought to be related to the presence of slowly repaired lesions. The authors concluded that the linear relationship between the dose rate is only present above a minimum dose rate.

In the most recent studies, the threshold for the onset of the FLASH effect is estimated to be 30Gy/s (47), 35Gy/s or 40Gy/s (48). For the data used in the present study, around 20% of the data points of all the FLASH studies use Mean Dose Rates below these thresholds. Therefore, it was considered that the dose rate may not be high enough for some data points for the FLASH effect to be observed. The combination of the data taken at dose rates above and below the FLASH threshold may dilute any observed linear correlation. It is therefore possible that some of the data included in the study lies below the FLASH threshold and that as a result no FLASH effect can be observed. Moreover, it is important to determine whether the FLASH threshold depends on other beam parameters such as particle type, total dose etc. To investigate the possible impact of a threshold, subsets of the data obtained at dose rates above 30Gy/s and above 40Gy/s were analysed separately (see **Supplementary Materials**). The trends observed were consistent with each other and with the trends observed in the full sample. This was equally apparent when examining **Figures 1**, **3**. There is no therefore evidence for a threshold effect in the data included in the present review study and all data have been retained in analyses presented above.

In order to demonstrate a full picture of all data, trends or thresholds for isolated parameters were not investigated in this review study. With no knowledge of a distribution, the linear regression analysis, confidence/prediction intervals and correlation coefficients are presented to look for an overarching trend rather than a study based on specific distributions. All data for trend testing, searching for potential thresholds (e.g. **Figure 1**) or removing statistical outliers for certain distributions that could drive correlations (e.g. **Figure 3**) can be found in the **Supplementary Materials**.

**Figure 6:**
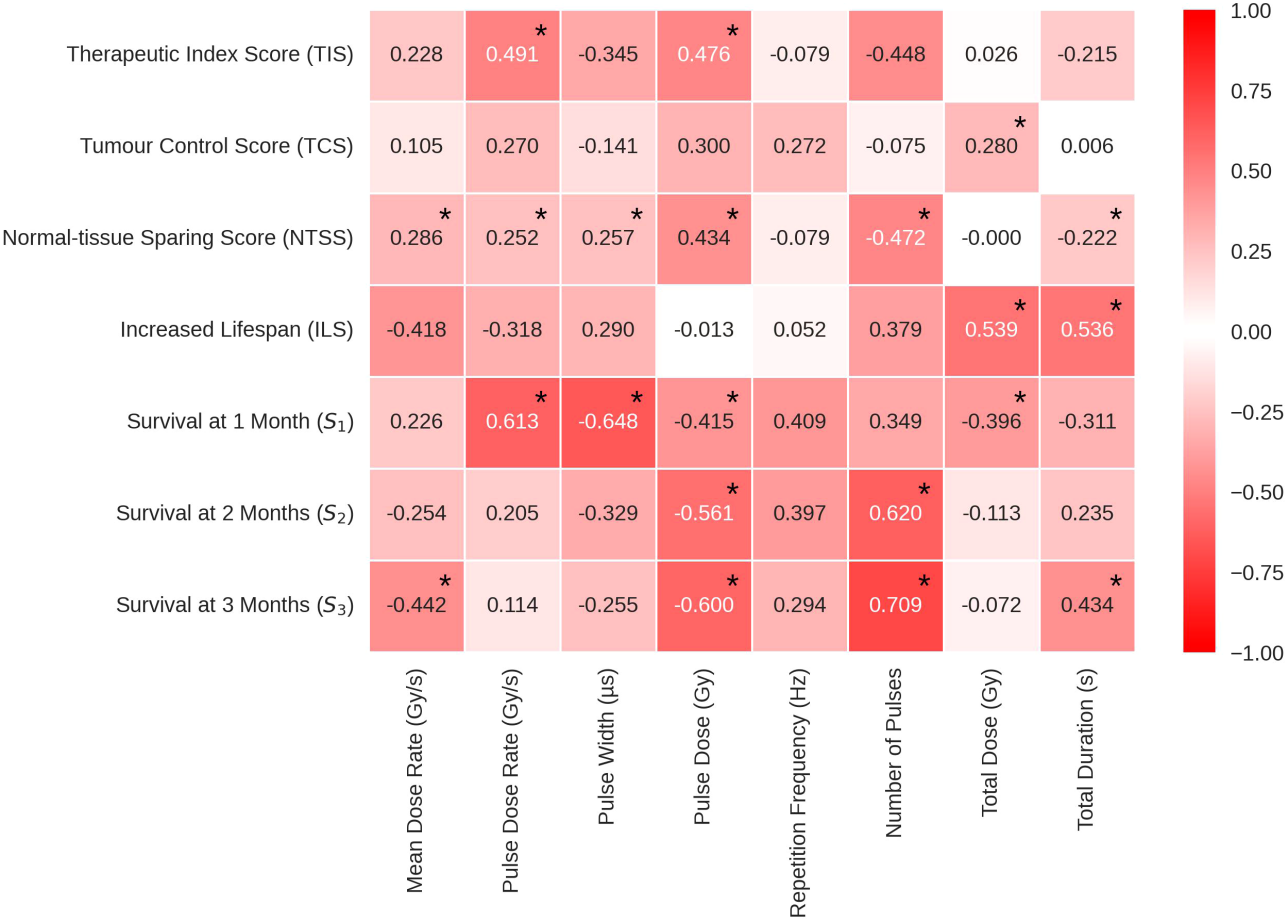
Pearson’s correlation coefficients in heat map form to show the correlations between the log of each beam parameter and the corresponding endpoint. The values range between -1 and 1, where the extremities (closest to -1 and 1) have the deepest colour and the weakest correlations (closer to 0) have a weak colour. Statistically significant correlations are identifiable by an asterisk at the top right of the corresponding correlation coefficient. TIS, Therapeutic Index Score; TCS, Tumour Control Score; NTSS, Normal-tissue Sparing Score; ILS, Increased Lifespan; *S*_1_, Survival % at 1 month; *S*_2_, Survival % at 2 month; *S*_3_, Survival % at 3 month.

An overview of the data is presented in **Figure 6**, where the Pearson correlation coefficients characterising the relationship between the logarithmic scored endpoints and the beam parameters are presented.

The cells in **Figure 6** are colour coded to indicate the strength of the correlation. The most significant TIS correlations were with Pulse Dose Rate and Pulse Dose, both positive, suggesting that a high pulse dose and dose rate increases therapeutic index. Pulse Dose Rate demonstrated positive correlations with nearly all endpoints, excluding ILS. This pattern suggests the potential viability of FLASH-RT as an effective and less toxic modality for radiotherapy. TCS and NTSS results suggest that beam parameter qualities from both FLASH and conventional radiotherapy can be useful to spare tissue and treat the tumour effectively.

While TCS correlations remained weak to moderate for all beam parameters, the correlation with Total Dose remains statistically significant. The correlation indicates that, in the data considered here, a larger dose administered to the tumour results in a higher level of tumour control and following a more classical model. Weak to moderate correlations are also observed for NTSS, having 5 statistically significant correlations. Thereby the FLASH effect showed statistically significant (although weak to moderate) correlations with Number of Pulses, Mean Dose Rate, Pulse Dose Rate, Pulse Dose and Total Duration. The NTSS correlations suggest that normal-tissue sparing in FLASH therapy can be favoured by both the use of short pulses with high dose rates *and* high doses with less pulses. These results suggest FLASH-RT could preserve tissue using a low amount of short, high dose pulses.

Negative correlations of ILS with Mean Dose Rate and Pulse Dose Rate are observed. Multivariate analyses carried out to investigate the correlation between the beam parameters displayed a strong positive relationships between these two parameters (see **Supplementary Materials**). This is expected, assuming most accelerators do not have extensive lapses between pulses. In addition, the ILS logged data shows statistically significant, strong positive correlations with Total Dose and Total Duration, suggesting that the parameters of tumour control weighted more in this case.

The percentage of survivals at short term (S_1_- 1 month post-RT) correlates positively with Pulse Dose Rate (the higher the pulse dose rate, the higher the percentage of survivals) and negatively with Pulse Width suggesting that these parameters are possibly related to the acute toxicity. This was not reproduced in the long term. For correlations with percentage of survivals at 3 months post-RT (S_3_), both Mean Dose Rate and Pulse Dose Rate held statistically significant negative correlations.

In addition, Pulse Dose Rate had a statistically significant negative correlation while Total Duration and Number of Pulses had a statistically significant positive correlation. In conjunction with the TCS scores, it appears that the survival difference long term (3 months post-RT) could be due to continuous beams having a more efficient tumour control. Observing the differences at 1 and 3 months post-RT, it appears that the variation in the time at which the endpoints are observed may be an additional source of inconsistency in the homogeneity of the manually scored data.

A recent study of the impact of FLASH-RT on glioblastoma investigated survival trends at 3 months (49). In order to compare the results of the present study with that presented in (49), glioblastoma studies were extracted from the S*_M_* database and survival was examined at 3 months. Survival at three months (S_3_) was averaged separately for ultrahigh dose rate irradiations (dose rate greater than 30Gy/s) and compared to the average S_3_ for conventional irradiations (CONV) of the same dose. It was found that the average survival at 3 months was very similar for FLASH and CONV, resulting in 54.8 ± 7.7% and 54.3 ± 7.6% respectively (see **Supplementary Materials**). This suggests that the survival response for this type of tumour is similar for both radiation modalities. Despite these results not being significant, the indication that FLASH may have a slightly higher response is similar to the conclusion drawn in the recently published study by Böhlen et al. (49).

## 4 Conclusion

The FLASH effect is thought to reduce the adverse toxicities during the radiation process. This review study presents an extensive analysis of experiments including investigations of FLASH-RT and its potential influences. A semi-quantitative approach was developed to assess each study, wherein the normal-tissue preservation and tumour-control capabilities were evaluated considering the outcomes of each experiment. The study establishes a correlation between ultra-high dose rates and the observed FLASH effect. This is evident through the significant associations found between Normal-tissue Sparing Score and Therapeutic Index Score with Pulse Dose Rate.

Additional data extrapolation was carried out to enable survival to be studied in order to evaluate the papers with a set endpoint. The Survival Score correlations are indicative of a short term sparing effect and a long term tumour control efficiency. This is seen in the data at 1-month, where both Mean Dose Rate and Pulse Dose Rate exhibit positive correlations and then observing how the relationship reverses for 2 and 3 months post FLASH-RT. This phenomenon suggests a delicate balance between normal-tissue sparing and effective tumour control. In addition, the Increased Lifespan data provides further support, indicating a significant positive correlation with both Total Dose and Total Duration. These findings underscore the need for higher doses and extended radiation times for comprehensive tumour treatment.

## Data availability statement

The original contributions presented in the study are included in the article/**Supplementary Material**. Further inquiries can be directed to the corresponding authors.

## Author contributions

JM: Writing – original draft, Writing – review & editing, Data curation, Formal analysis, Methodology. KL: Writing – original draft, Writing – review & editing, Supervision, Validation. YP: Writing – original draft, Writing – review & editing, Supervision.
