# Supplementary material for: The FLASH effect—an evaluation of preclinical studies of ultra-high dose rate radiotherapy
Source: Front Oncol. 2024 Apr 22;14:1340190. doi: 10.3389/fonc.2024.1340190 (PMC11071325; doi:10.3389/fonc.2024.1340190)
Supplement: Supplementary file 1 [file Presentation_1.pdf]

The FLASH effect—An evaluation of preclinical studies of ultra-high dose rate radiotherapy: Supplementary Materials

Contents

1 Additional Graphs (not in main body) 2

Pearson's Correlation Coefficients in heat map form to show the correlations between each dosimetric parameter and the corresponding endpoint 2

Pearson's Correlation Coefficients in heat map form to show the correlations between the log of each dosimetric parameter and the corresponding endpoint for all data with mean and pulse dose rates above 30Gy/s. 3

Pearson's Correlation Coefficients in heat map form to show the correlations between the log of each dosimetric parameter and the corresponding endpoint for all data with mean and pulse dose rates above 40Gy/s. 4

Pearson's Correlation Coefficients in heat map form to show the correlations between the each dosimetric parameter. 5

2 Full Versions of Graphs (in main body) 6

Pearson's Correlation Coefficients in heat map form to show the correlations between the log of each dosimetric parameter and the corresponding endpoint. 6

TIS plotted against the strongest dosimetric parameter, Total Dose. 7

TCS plotted against the strongest dosimetric parameter, Total Time. 8

NTSS plotted against the strongest dosimetric parameter, Pulse Dose Rate. 9

ILS plotted against the strongest dosimetric parameter, Total Dose. 10

SS percentage plotted against the strongest dosimetric parameter, Number of Pulses. 11

3 Data Tables 12

Full table of beam parameters including the title of each study with the corresponding Increased Lifespan (ILS). 12

Full table of beam parameters including the title of each study with the corresponding % survivals (S_M). 13

Full table of beam parameters including the title of each study with the corresponding TIS, TCS and NTSS. 14

% survivors of Glioma-bearing rats at 3 months post FLASH vs CONV. 15

% Alignment of each normal-tissue experiment with the PICO search strategy. 16

% Alignment of each tumour experiment with the PICO search strategy. 18

1 Additional Graphs (not in main body)

Figure 1: Pearson's Correlation Coefficients in heat map form to show the correlations between each dosimetric parameter and the corresponding endpoint **over a non-logarithmic scale**. The values range between -1 and 1, where the extremities (closest to -1 and 1) have the deepest colour and the weakest correlations (closer to 0) have a weak colour. Statistically significant correlations are identifiable by an asterisk at the top right of the corresponding correlation coefficient. Key: TIS- Therapeutic Index Score, TCS- Tumour Control Score, NTSS- Normal-tissue Sparing Score, ILS- Increased Lifespan, S_1- Survival % at 1 month, S_2- Survival % at 2 month, S_3- Survival % at 3 month.

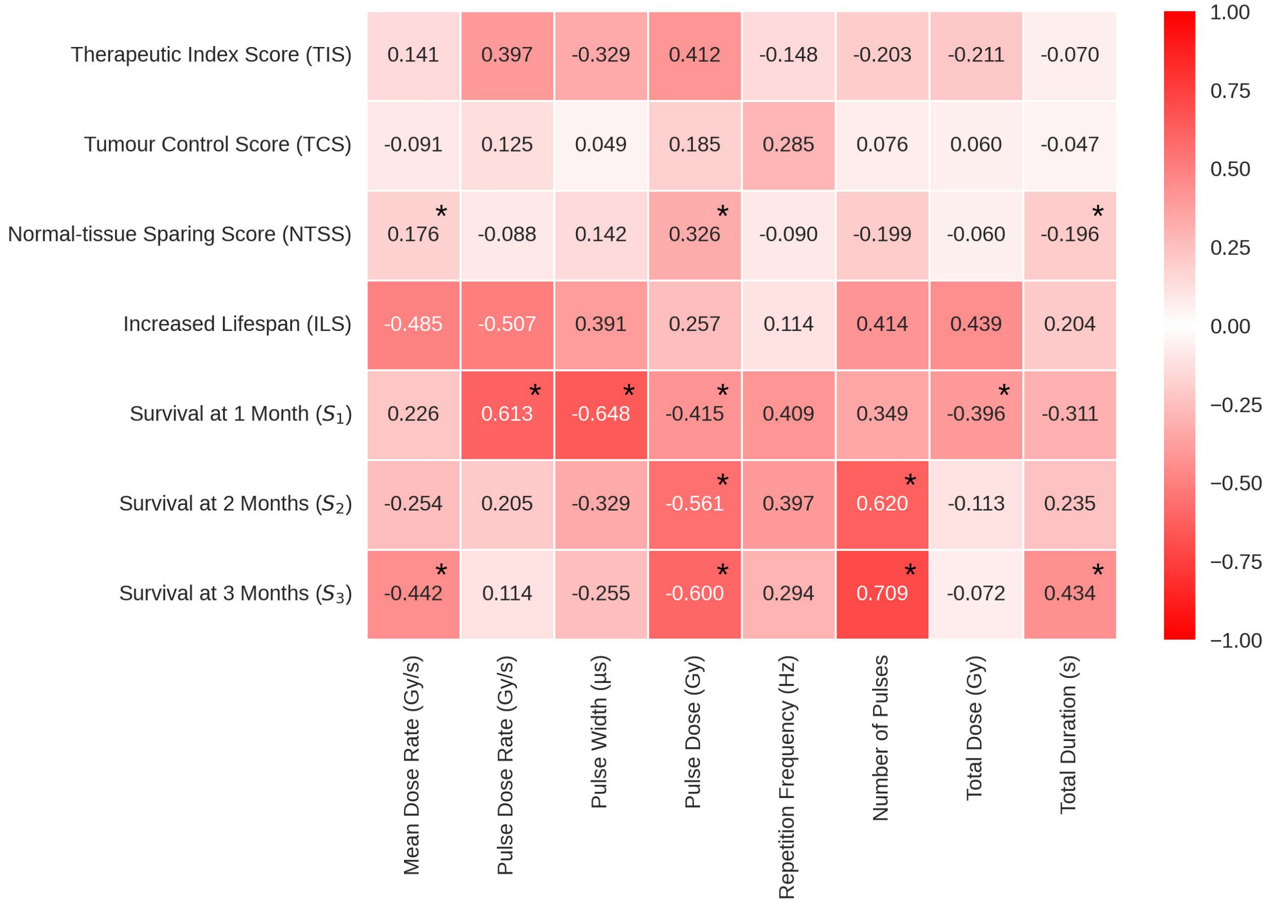

Figure 2: Pearson's Correlation Coefficients in heat map form to show the correlations between the log of each dosimetric parameter and the corresponding endpoint **for all data with mean and pulse dose rates above 30Gy/s.** The values range between -1 and 1, where the extremities (closest to -1 and 1) have the deepest colour and the weakest correlations (closer to 0) have a weak colour. Statistically significant correlations are identifiable by an asterisk at the top right of the corresponding correlation coefficient. Key: TIS- Therapeutic Index Score, TCS- Tumour Control Score, NTSS- Normal-tissue Sparing Score, ILS- Increased Lifespan, S_1- Survival % at 1 month, S_2- Survival % at 2 month, S_3- Survival % at 3 month.

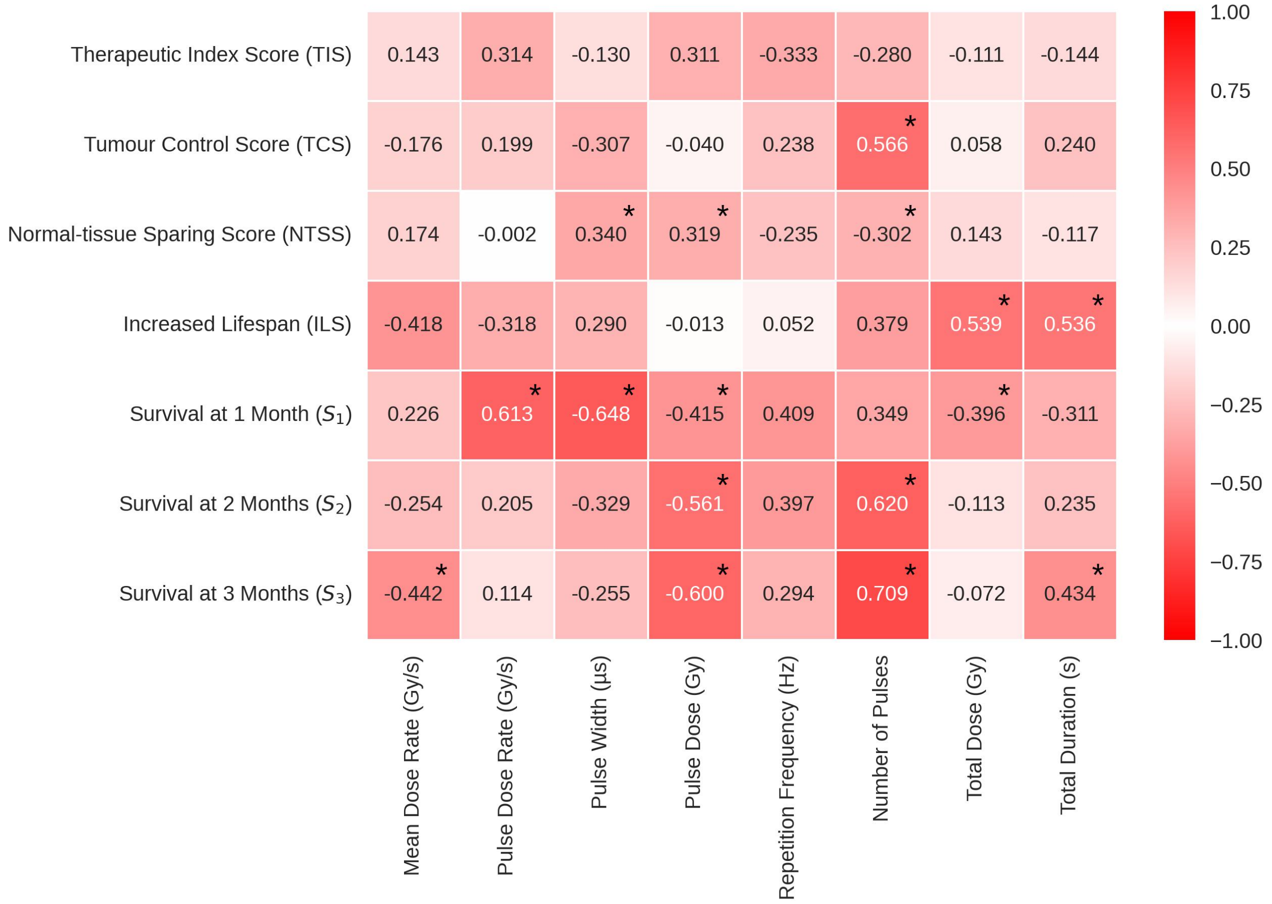

Figure 3: Pearson's Correlation Coefficients in heat map form to show the correlations between the log of each dosimetric parameter and the corresponding endpoint **for all data with mean and pulse dose rates above 40Gy/s.** The values range between -1 and 1, where the extremities (closest to -1 and 1) have the deepest colour and the weakest correlations (closer to 0) have a weak colour. Statistically significant correlations are identifiable by an asterisk at the top right of the corresponding correlation coefficient. Key: TIS- Therapeutic Index Score, TCS- Tumour Control Score, NTSS- Normal-tissue Sparing Score, ILS- Increased Lifespan, S_1- Survival % at 1 month, S_2- Survival % at 2 month, S_3- Survival % at 3 month.

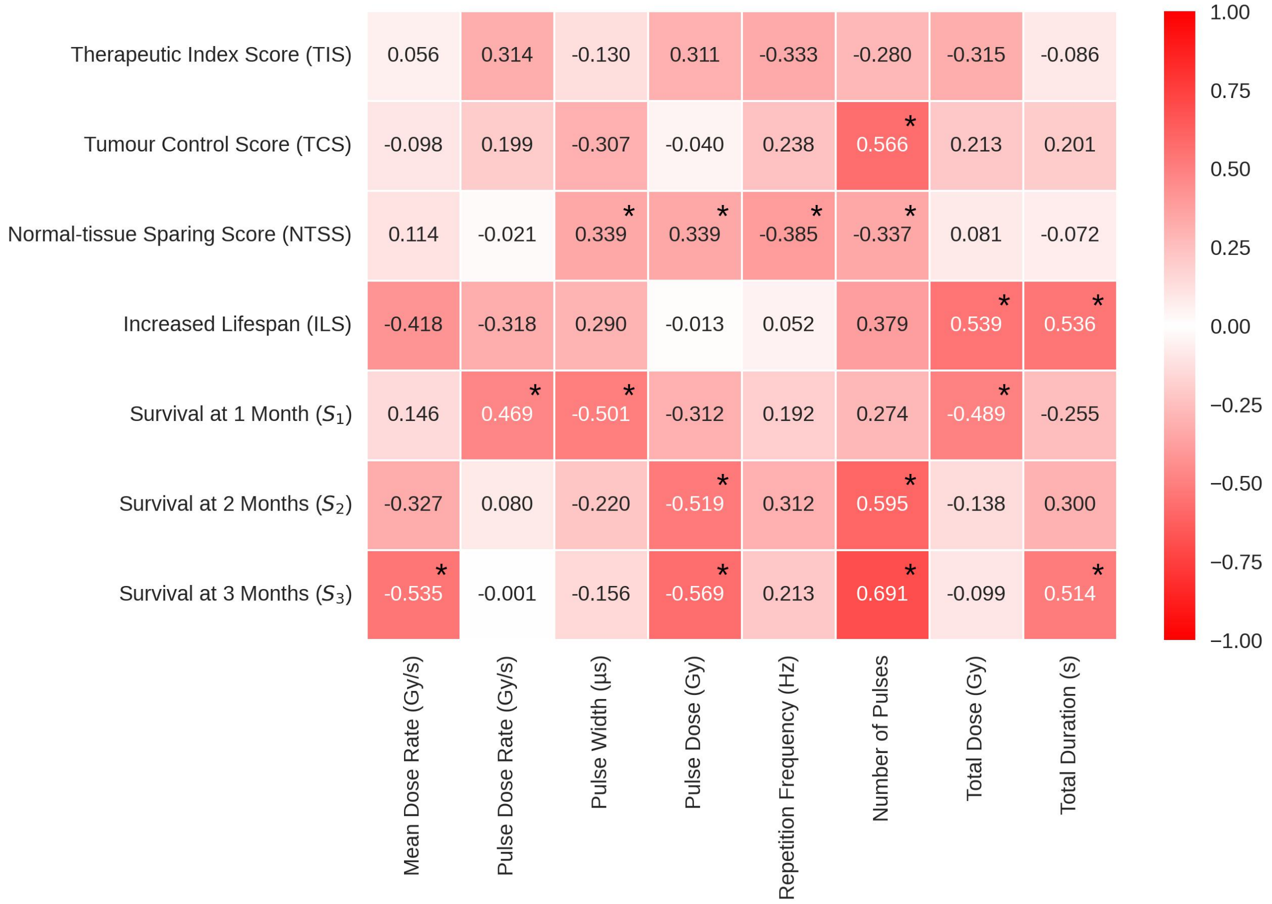

Figure 4: Pearson's Correlation Coefficients in heat map form to show the correlations between the each dosimetric parameter. The values range between -1 and 1, where the extremities (closest to -1 and 1) have the deepest colour and the weakest correlations (closer to 0) have a weak colour.

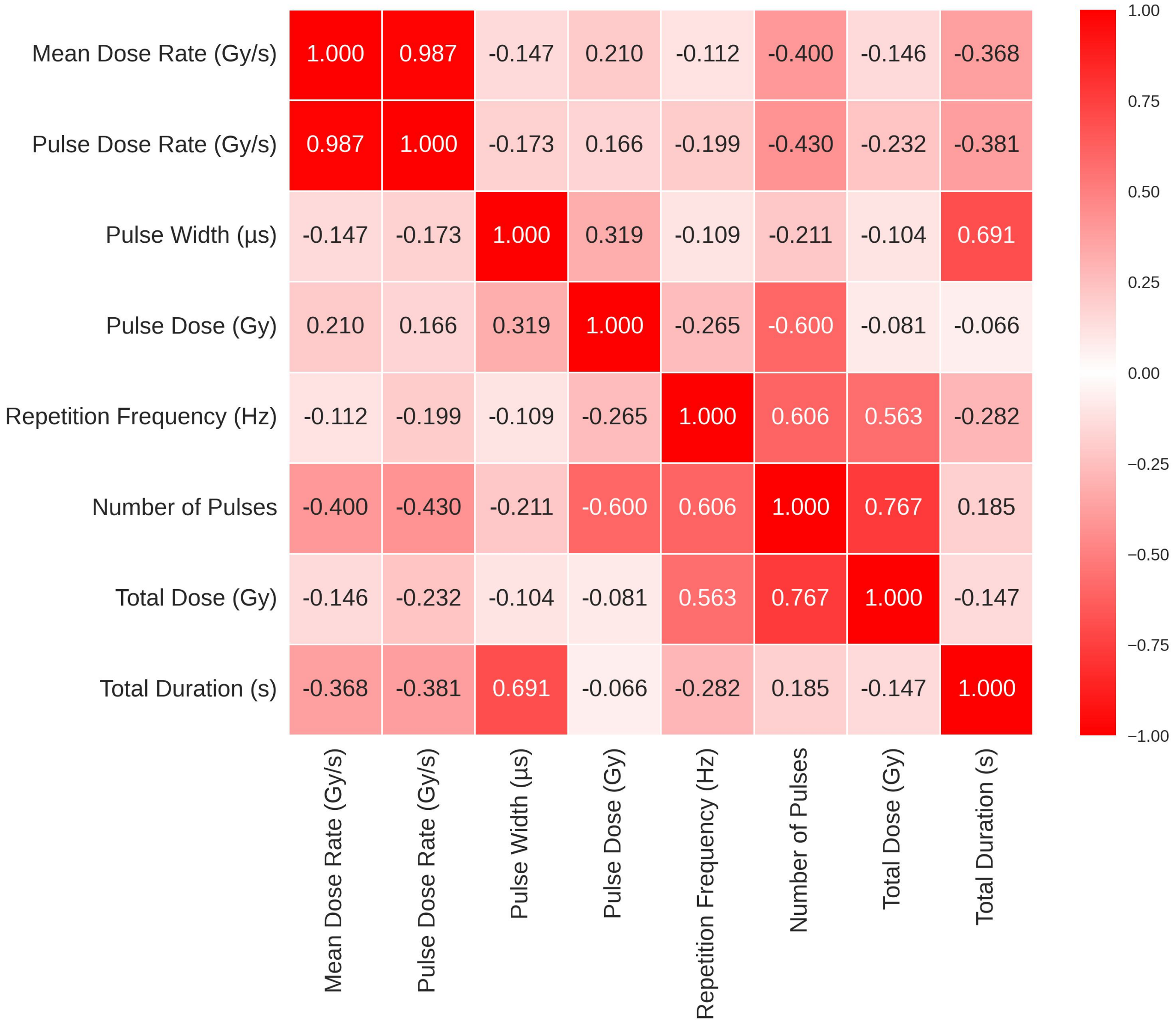

2 Full Versions of Graphs (in main body)

Figure 5: Pearson's Correlation Coefficients in heat map form to show the correlations between the log of each dosimetric parameter and the corresponding endpoint. The values range between -1 and 1, where the extremities (closest to -1 and 1) have the deepest colour and the weakest correlations (closer to 0) have a weak colour. Statistically significant correlations are identifiable by an asterisk at the top right of the corresponding correlation coefficient. Key: TIS- Therapeutic Index Score, TCS- Tumour Control Score, NTSS- Normal-tissue Sparing Score, ILS- Increased Lifespan, S_1- Survival % at 1 month, S_2- Survival % at 2 month, S_3- Survival % at 3 month.

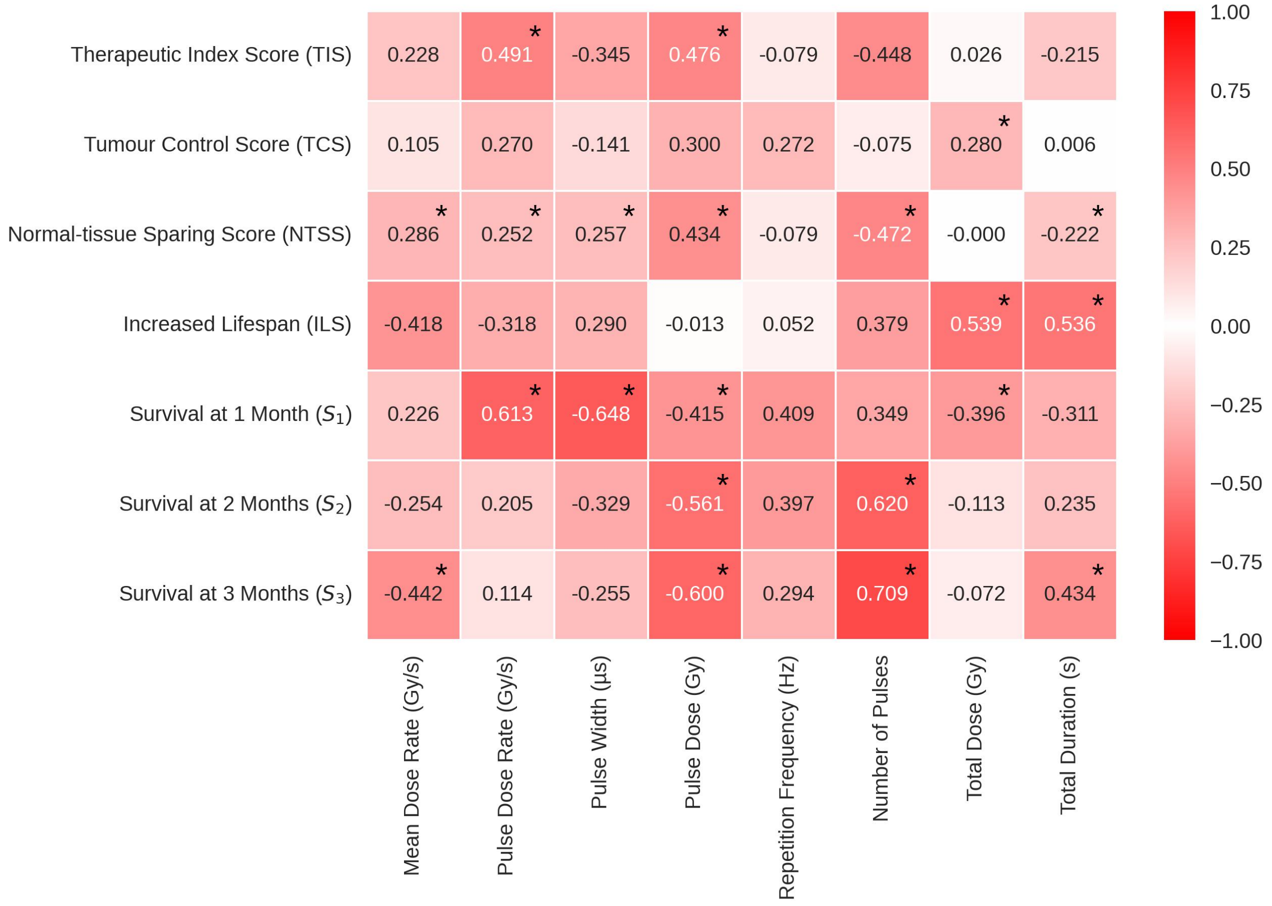

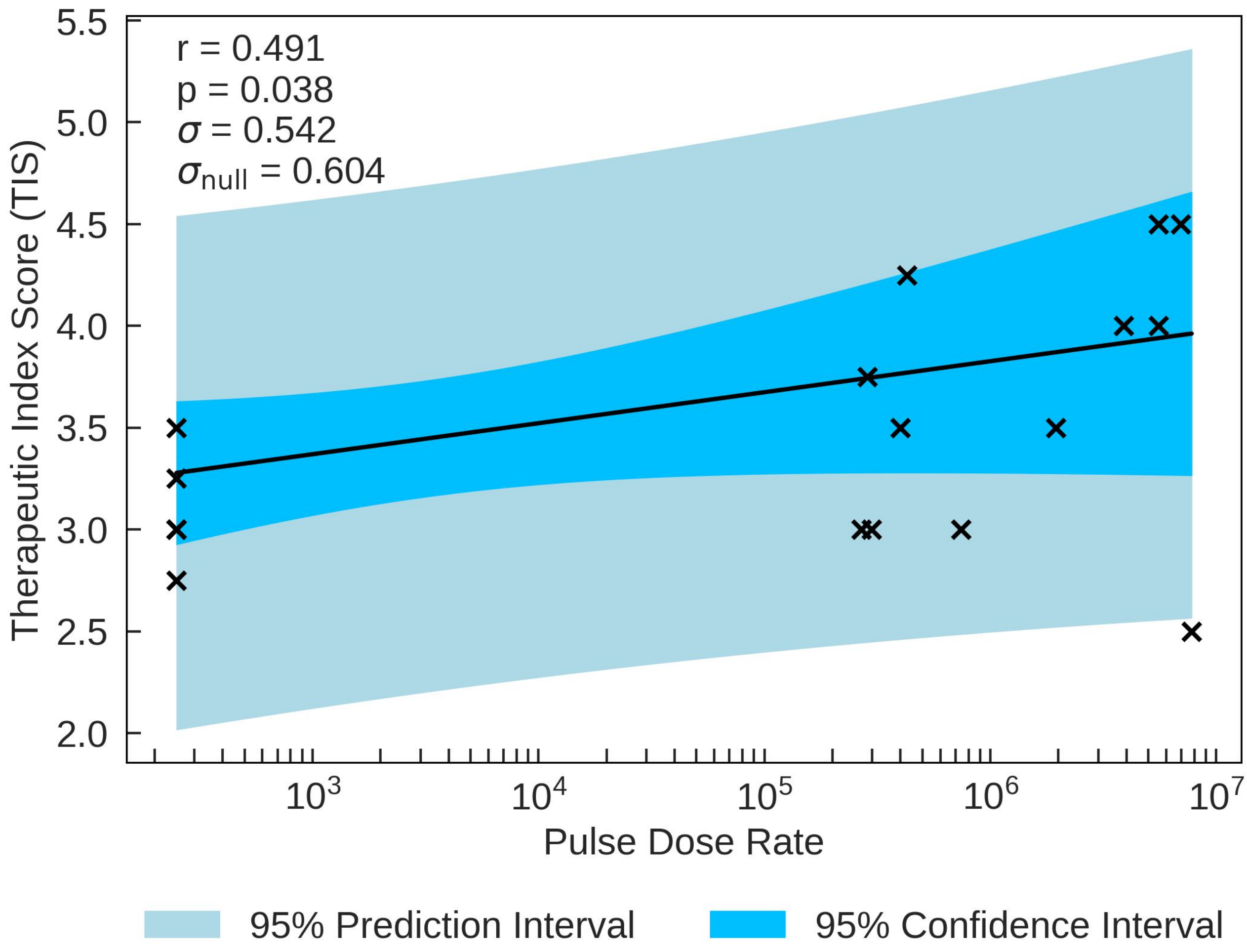

Figure 6: TIS plotted against the strongest dosimetric parameter, Pulse Dose Rate. There is a moderate to strong positive correlation between the parameters, showing that an increase in dose rate will increase the chance of observing a higher therapeutic index.

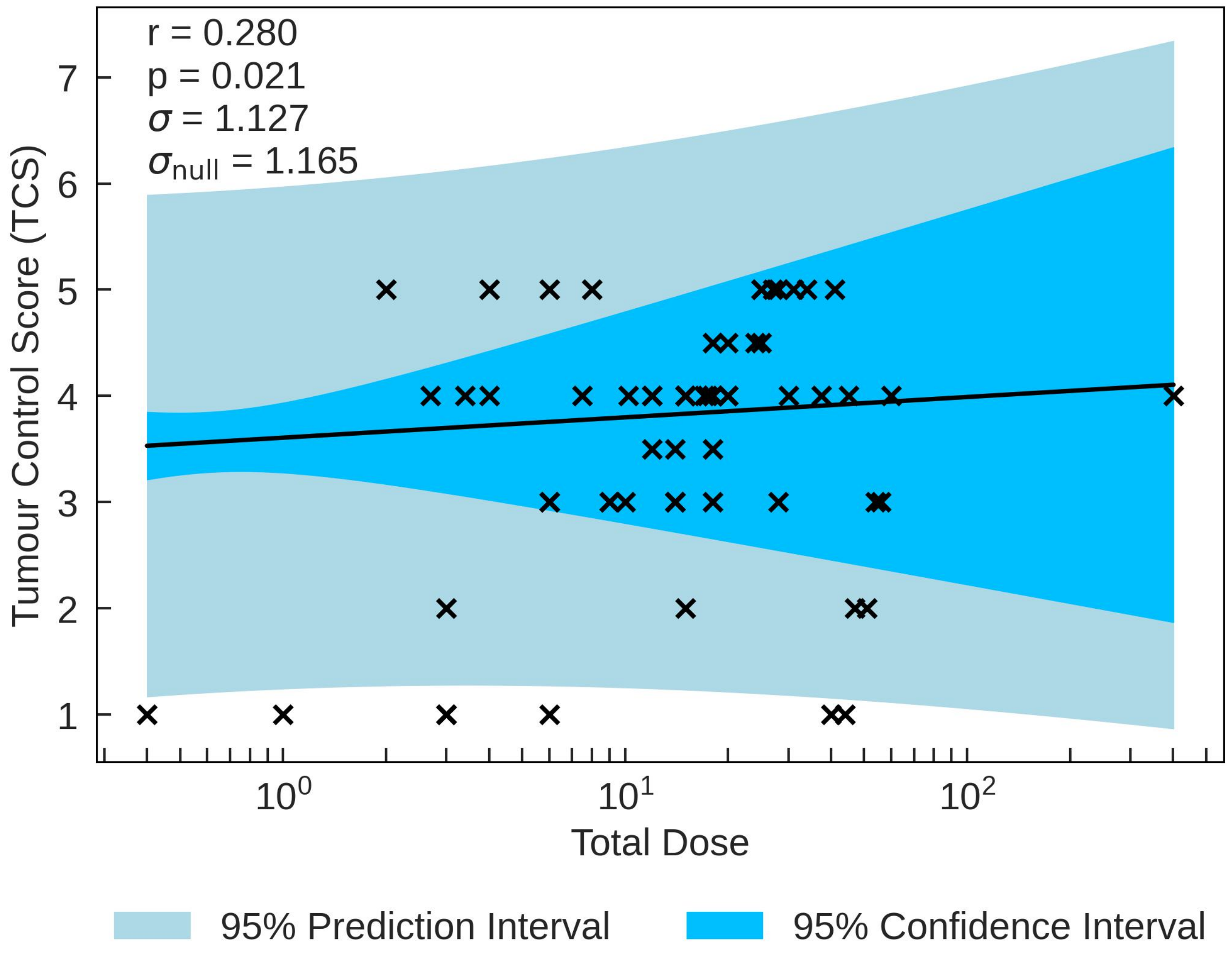

Figure 7: TCS plotted against the strongest dosimetric parameter, Total Dose. There is a moderate positive correlation between the parameters, suggesting that an increase in irradiation dose may increase tumour control.

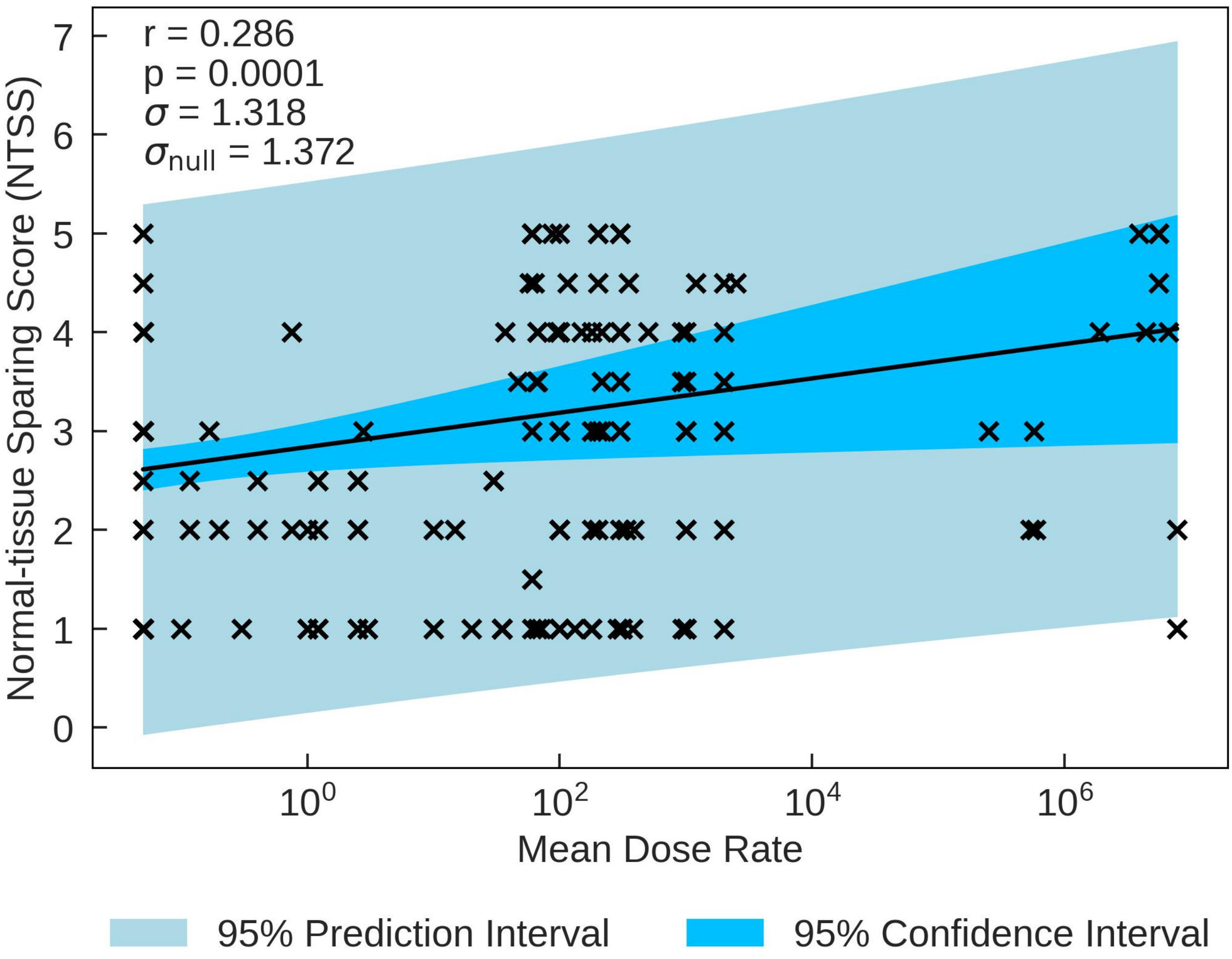

Figure 8: NTSS plotted against the strongest dosimetric parameter, Mean Dose Rate. There is a moderate positive correlation between the parameters, suggesting that an increase in the overall dose rate may increase the chance of observing a sparing effect in normal tissue (observation of the FLASH effect).

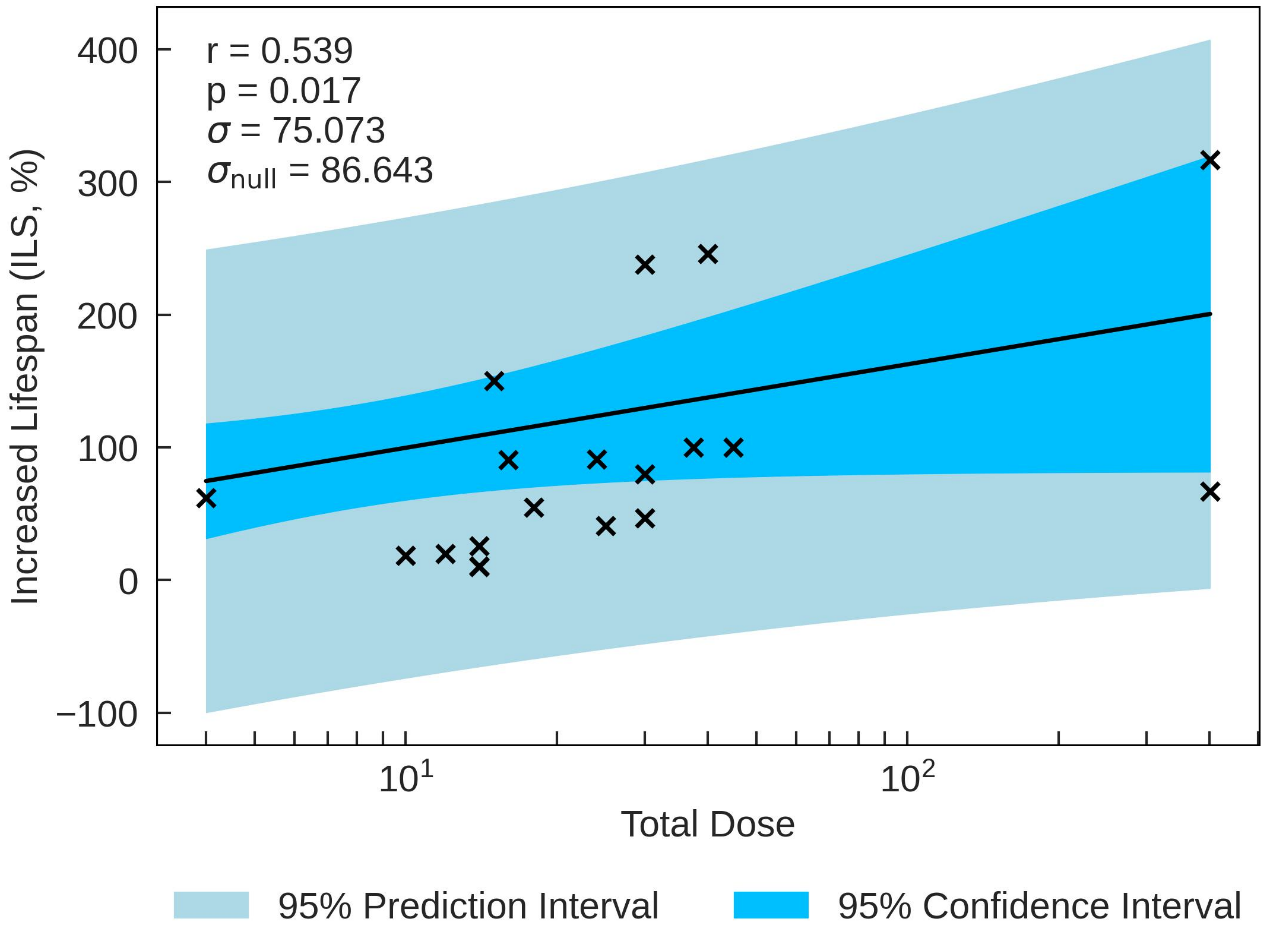

Figure 9: ILS plotted against the strongest dosimetric parameter, Total Dose. There is a strong positive correlation between the parameters, illustrating that an increase in dose can increase the lifespan of small animals.

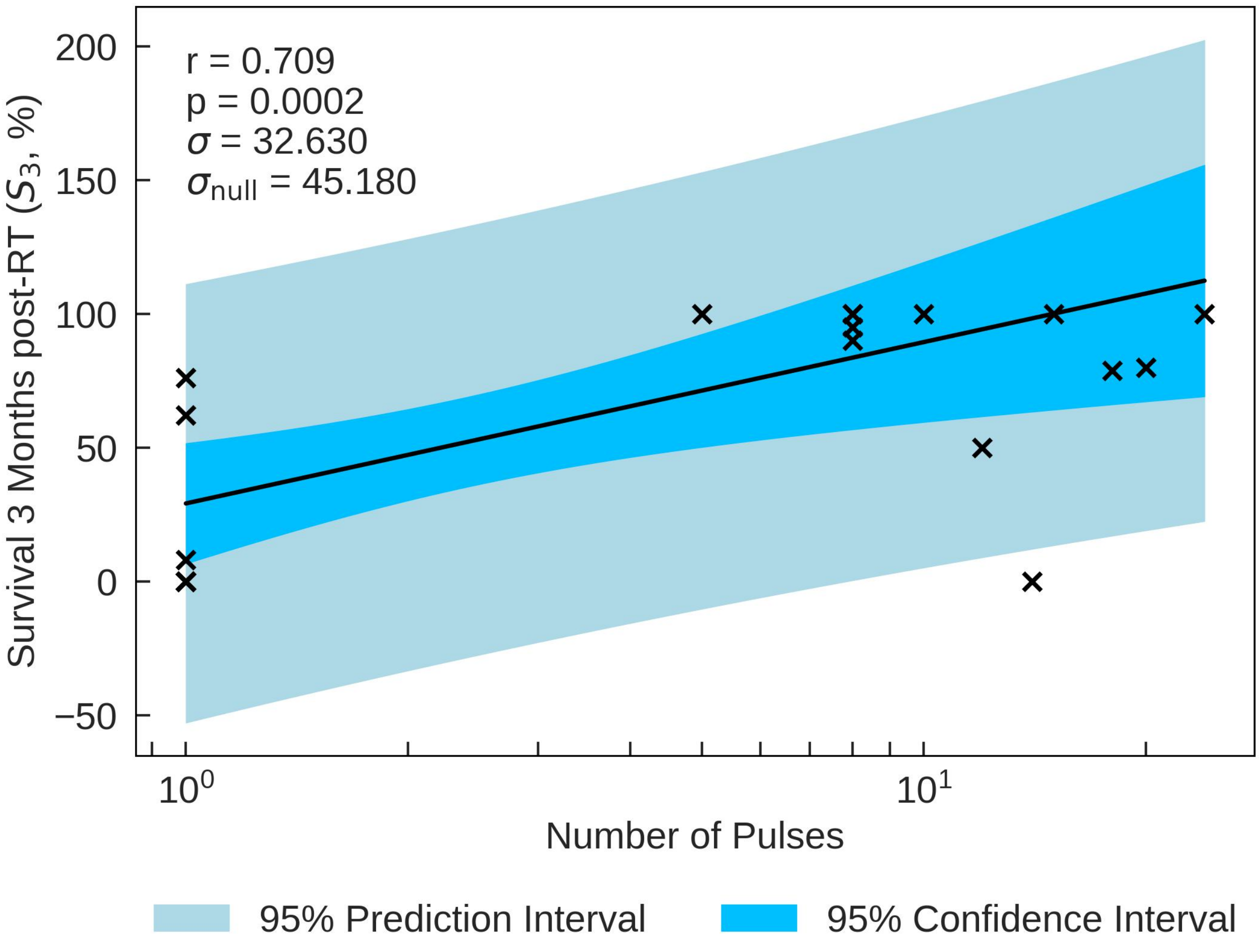

Figure 10: SS percentage plotted against the strongest dosimetric parameter, Number of Pulses. There is a strong positive correlation between the parameters, illustrating that an increase in the Number of Pulses can increase the survival time of small animals.

3 Data Tables

Table 1: Full table of beam parameters including the title of each study with the corresponding Increased Lifespan (ILS).

Title	Mean Dose Rate (Gy/s)	Pulse Dose Rate (Gy/s)	Pulse Width (µs)	Pulse Dose (Gy)	Repetition Frequency (Hz)	Number of Pulses	Total Dose (Gy)	Total Duration (s)	Energy (MeV)	ILS
Complete Remission of Mouse Melanoma after Temporally Fractionated Microbeam Radiotherapy	1.29E+04						4.01E+02	3.11E-02	1.04E-01	6.67E+0
Complete Remission of Mouse Melanoma after Temporally Fractionated Microbeam Radiotherapy	1.17E+04						4.01E+02	3.43E-02	1.04E-01	3.17E+0
Hypofractionated FLASH-RT as an Effective Treatment against Glioblastoma that Reduces Neurocognitive Side Effects in Mice	5.60E+06	5.56E+06	1.80E+00	1.00E+01	1.00E+02	1.00E+00	1.00E+01	1.80E-06	6.00E+00	1.85E+0
Hypofractionated FLASH-RT as an Effective Treatment against Glioblastoma that Reduces Neurocognitive Side Effects in Mice	7.80E+06	7.78E+06	1.80E+00	1.40E+01	1.00E+02	1.00E+00	1.40E+01	1.80E-06	6.00E+00	2.58E+0
Hypofractionated FLASH-RT as an Effective Treatment against Glioblastoma that Reduces Neurocognitive Side Effects in Mice	1.90E+06	1.94E+06	1.80E+00	3.50E+00	1.00E+02	1.00E+00	4 x 3.50E+00	1.80E-06	6.00E+00	1.06E+0
Hypofractionated FLASH-RT as an Effective Treatment against Glioblastoma that Reduces Neurocognitive Side Effects in Mice	3.90E+06	3.89E+06	1.80E+00	7.00E+00	1.00E+02	1.00E+00	2 x 7.00E+00	1.80E-06	6.00E+00	9.77E+0
Hypofractionated FLASH-RT as an Effective Treatment against Glioblastoma that Reduces Neurocognitive Side Effects in Mice	5.60E+06	5.56E+06	1.80E+00	1.00E+01	1.00E+02	1.00E+00	3 x 1.00E+01	1.80E-06	6.00E+00	4.66E+0
First demonstration of the FLASH effect with ultrahigh dose-rate high-energy X-rays	1.00E+03	1.00E+03	1.80E+04	1.80E+01	5.56E+01	1.00E+00	1.80E+01	1.80E-02	8.00E+00	5.49E+0
First demonstration of the FLASH effect with ultrahigh dose-rate high-energy X-rays	7.00E+02	7.00E+02	4.29E+04	3.00E+01	2.33E+01	1.00E+00	3.00E+01	4.29E-02	8.00E+00	2.38E+0
Ultra-high-dose-rate FLASH and Conventional-Dose-Rate Irradiation Differentially Affect Human Acute Lymphoblastic Leukemia and Normal Hematopoiesis	2.00E+02	1.11E+06	1.80E+00	2.00E+00	1.00E+02	2.00E+00	4.00E+00	2.00E-02	6.00E+00	6.20E+0
Comparable Long-Term Tumor Control for Hypofractionated FLASH Versus Conventional Radiation Therapy in an Immunocompetent Rat Glioma Model	5.71E+05	2.86E+05	3.50E+00	2.00E+00	2.86E+05	4 x 3.00E+00	8 x 3.00E+00	4.20E-05	1.00E+01	9.10E+0
Comparable Long-Term Tumor Control for Hypofractionated FLASH Versus Conventional Radiation Therapy in an Immunocompetent Rat Glioma Model	5.95E+05	2.98E+05	3.50E+00	2.08E+00	2.86E+05	6 x 3.00E+00	12.5 x 3.00E+00	6.30E-05	1.00E+01	1.00E+0
Comparable Long-Term Tumor Control for Hypofractionated FLASH Versus Conventional Radiation Therapy in an Immunocompetent Rat Glioma Model	5.36E+05	2.68E+05	3.50E+00	1.88E+00	2.86E+05	8 x 3.00E+00	15 x 3.00E+00	8.40E-05	1.00E+01	1.00E+0
Long-term anti-tumor effects following both conventional radiotherapy and FLASH in fully immunocompetent animals with glioblastoma	6.60E+01	1.33E+02	3.00E+04	4.00E+00	3.33E+01	2 x 4.00E+00	2 x 8.00E+00	2 x 1.20E-01		9.05E+0
Long-term anti-tumor effects following both conventional radiotherapy and FLASH in fully immunocompetent animals with glioblastoma	7.40E+01	7.35E+01	2.43E+04	1.79E+00	3.33E+01	2 x 7.00E+00	2 x 1.25E+01	2 x 1.70E-01		4.09E+0
FLASH Irradiation Results in Reduced Severe Skin Toxicity Compared to Conventional-Dose-Rate Irradiation	1.80E+02	4.00E+05	5.00E+00	2.00E+00	9.00E+01	1.50E+01	3.00E+01	1.67E-01	1.60E+01	8.00E+0
FLASH Irradiation Results in Reduced Severe Skin Toxicity Compared to Conventional-Dose-Rate Irradiation	1.80E+02	4.00E+05	5.00E+00	2.00E+00	9.00E+01	2.00E+01	4.00E+01	2.22E-01	1.60E+01	2.46E+0
Ultrahigh dose-rate FLASH irradiation increases the differential response between normal and tumor tissue in mice	6.00E+01						1.50E+01	2.50E-01	4.50E+00	1.50E+0
Antitumor Effect by Either FLASH or Conventional Dose Rate Irradiation Involves Equivalent Immune Responses							2 x 6.00E+00			2.00E+0
	**Key:**	Black text	From paper							
		Red text	Calculated							

Table 2: Full table of beam parameters including the title of each study with the corresponding % survivals (S_M).

Title	Mean Dose Rate (Gy/s)	Pulse Dose Rate (Gy/s)	Pulse Width (µs)	Pulse Dose (Gy)	Repetition Frequency (Hz)	Number of Pulses	Total Dose (Gy)	Total Duration (s)	Energy (MeV)	One month survival %	Two month survival %	Three month survival %	Six month survival %
Complete Remission of Mouse Melanoma after Temporally Fractionated Microbeam Radiotherapy	1.29E+04						4.01E+02	3.11E-02	1.04E-01	0.00E+00	0.00E+00	0.00E+00	0.00E+00
Complete Remission of Mouse Melanoma after Temporally Fractionated Microbeam Radiotherapy	1.17E+04						4.01E+02	3.43E-02	1.04E-01	3.50E+01	3.50E+01	3.50E+01	3.50E+01
FLASH irradiation enhances the therapeutic index of abdominal radiotherapy for the treatment of ovarian cancer	2.16E+02	1.00E+06	2.00E+00	2.00E+00	1.08E+02	8.00E+00	1.60E+01	7.41E-02	1.60E+01	9.50E+01	9.50E+01	9.50E+01	
Hypofractionated FLASH-RT as an Effective Treatment against Glioblastoma that Reduces Neurocognitive Side Effects in Mice	5.60E+06	5.56E+06	1.80E+00	1.00E+01	1.00E+02	1.00E+00	1.00E+01	1.80E-06	6.00E+00	1.00E+02	0.00E+00	0.00E+00	0.00E+00
Hypofractionated FLASH-RT as an Effective Treatment against Glioblastoma that Reduces Neurocognitive Side Effects in Mice	7.80E+06	7.78E+06	1.80E+00	1.40E+01	1.00E+02	1.00E+00	1.40E+01	1.80E-06	6.00E+00	1.00E+02	0.00E+00	0.00E+00	0.00E+00
Hypofractionated FLASH-RT as an Effective Treatment against Glioblastoma that Reduces Neurocognitive Side Effects in Mice	1.90E+06	1.94E+06	1.80E+00	3.50E+00	1.00E+02	1.00E+00	4 x 3.50E+00	1.80E-06	6.00E+00	8.30E+01	8.00E+00	0.00E+00	0.00E+00
Hypofractionated FLASH-RT as an Effective Treatment against Glioblastoma that Reduces Neurocognitive Side Effects in Mice	3.90E+06	3.89E+06	1.80E+00	7.00E+00	1.00E+02	1.00E+00	2 x 7.00E+00	1.80E-06	6.00E+00	1.00E+02	0.00E+00	0.00E+00	0.00E+00
Hypofractionated FLASH-RT as an Effective Treatment against Glioblastoma that Reduces Neurocognitive Side Effects in Mice	5.60E+06	5.56E+06	1.80E+00	1.00E+01	1.00E+02	1.00E+00	3 x 1.00E+01	1.80E-06	6.00E+00	1.00E+02	8.00E+01	8.00E+00	0.00E+00
First demonstration of the FLASH effect with ultrahigh dose-rate high-energy X-rays	1.00E+03	1.00E+03	1.80E+04	1.80E+01	5.56E+01	1.00E+00	1.80E+01	1.80E-02	8.00E+00	1.00E+02	2.00E+01	0.00E+00	0.00E+00
First demonstration of the FLASH effect with ultrahigh dose-rate high-energy X-rays	1.20E+03	1.20E+03	2.50E+04	3.00E+01	4.00E+01	1.00E+00	3.00E+01	2.50E-02	8.00E+00	9.00E+01	9.00E+01	7.60E+01	0.00E+00
First demonstration of the FLASH effect with ultrahigh dose-rate high-energy X-rays	9.37E+02	9.37E+02	1.60E+04	1.50E+01	6.25E+01	1.00E+00	1.50E+01	1.60E-02	8.00E+00	0.00E+00	0.00E+00	0.00E+00	0.00E+00
First demonstration of the FLASH effect with ultrahigh dose-rate high-energy X-rays				1.20E+01		1.00E+00	1.20E+01		8.00E+00	6.20E+01	6.20E+01	6.20E+01	
Ultra-high-dose-rate FLASH and Conventional-Dose-Rate Irradiation Differentially Affect Human Acute Lymphoblastic Leukemia and Normal Hematopoiesis	2.00E+02	1.11E+06	1.80E+00	2.00E+00	1.00E+02	2.00E+00	4.00E+00	2.00E-02	6.00E+00	1.00E+02	1.00E+02		
Ultra high dose rate (35 Gy/sec) radiation does not spare the normal tissue in cardiac and splenic models of lymphopenia and gastrointestinal syndrome	3.50E+01	3.50E+01	4.57E+05	1.60E+01	4.57E-01	1.00E+00	1.60E+01	4.57E-01	2.00E+01	0.00E+00	0.00E+00	0.00E+00	0.00E+00
Comparable Long-Term Tumor Control for Hypofractionated FLASH Versus Conventional Radiation Therapy in an Immunocompetent Rat Glioma Model	5.71E+05	2.86E+05	3.50E+00	2.00E+00	2.86E+05	4 x 3.00E+00	8 x 3.00E+00	4.20E-05	1.00E+01	1.00E+02	1.00E+02	5.00E+01	
Comparable Long-Term Tumor Control for Hypofractionated FLASH Versus Conventional Radiation Therapy in an Immunocompetent Rat Glioma Model	5.95E+05	2.98E+05	3.50E+00	2.08E+00	2.86E+05	6 x 3.00E+00	12.5 x 3.00E+00	6.30E-05	1.00E+01	1.00E+02	9.00E+01	7.88E+01	
Comparable Long-Term Tumor Control for Hypofractionated FLASH Versus Conventional Radiation Therapy in an Immunocompetent Rat Glioma Model	5.36E+05	2.68E+05	3.50E+00	1.88E+00	2.86E+05	8 x 3.00E+00	15 x 3.00E+00	8.40E-05	1.00E+01	1.00E+02	1.00E+02	1.00E+02	
Long-term anti-tumor effects following both conventional radiotherapy and FLASH in fully immunocompetent animals with glioblastoma	6.60E+01	1.33E+02	3.00E+04	4.00E+00	3.33E+01	2 x 4.00E+00	2 x 8.00E+00	2 x 1.20E-01		1.00E+02	1.00E+02	1.00E+02	
Long-term anti-tumor effects following both conventional radiotherapy and FLASH in fully immunocompetent animals with glioblastoma	7.40E+01	7.35E+01	2.43E+04	1.79E+00	3.33E+01	2 x 7.00E+00	2 x 1.25E+01	2 x 1.70E-01		6.40E+01	0.00E+00	0.00E+00	0.00E+00
FLASH Irradiation Results in Reduced Severe Skin Toxicity Compared to Conventional-Dose-Rate Irradiation	1.80E+02	4.00E+05	5.00E+00	2.00E+00	9.00E+01	5.00E+00	1.00E+01	5.56E-02	1.60E+01	1.00E+02	1.00E+02	1.00E+02	1.00E+02
FLASH Irradiation Results in Reduced Severe Skin Toxicity Compared to Conventional-Dose-Rate Irradiation	1.80E+02	4.00E+05	5.00E+00	2.00E+00	9.00E+01	8.00E+00	1.60E+01	8.89E-02	1.60E+01	1.00E+02	1.00E+02	1.00E+02	1.00E+02
FLASH Irradiation Results in Reduced Severe Skin Toxicity Compared to Conventional-Dose-Rate Irradiation	1.80E+02	4.00E+05	5.00E+00	2.00E+00	9.00E+01	1.00E+01	2.00E+01	1.11E-01	1.60E+01	1.00E+02	1.00E+02	1.00E+02	1.00E+02
FLASH Irradiation Results in Reduced Severe Skin Toxicity Compared to Conventional-Dose-Rate Irradiation	1.80E+02	4.00E+05	5.00E+00	2.00E+00	9.00E+01	1.50E+01	3.00E+01	1.67E-01	1.60E+01	1.00E+02	1.00E+02	1.00E+02	1.00E+02
FLASH Irradiation Results in Reduced Severe Skin Toxicity Compared to Conventional-Dose-Rate Irradiation	1.80E+02	4.00E+05	5.00E+00	2.00E+00	9.00E+01	2.00E+01	4.00E+01	2.22E-01	1.60E+01	1.00E+02	8.00E+01	8.00E+01	5.30E+01
Ultrahigh dose-rate FLASH irradiation increases the differential response between normal and tumor tissue in mice	6.00E+01						1.50E+01	2.50E-01	4.50E+00	4.00E+01	2.00E+01		
Ultrahigh dose-rate FLASH irradiation increases the differential response between normal and tumor tissue in mice	6.00E+01						2.30E+01	3.83E-01	4.50E+00	8.00E+01	6.00E+01		
Ultrahigh dose-rate FLASH irradiation increases the differential response between normal and tumor tissue in mice	6.00E+01						2.80E+01	4.67E-01	4.50E+00	8.90E+01	6.00E+01		
Abdominal FLASH irradiation reduces radiation-induced gastrointestinal toxicity for the treatment of ovarian cancer in mice	2.16E+02	4.00E+05	5.00E+00	2.00E+00	1.08E+02	8.00E+00	1.60E+01	7.41E-02	1.60E+01	9.00E+01	9.00E+01	9.00E+01	

Key: Black text — From paper; Red text — Calculated

Table 3: Full table of beam parameters including the title of each study with the corresponding TIS, TCS and NTSS.

Title	Mean Dose Rate (Gy/s)	Pulse Dose Rate (Gy/s)	Pulse Width (µs)	Pulse Dose (Gy)	Repetition Frequency (Hz)	Number of Pulses	Total Dose (Gy)	Total Duration (s)	Accelerator	Particle	Energy (MeV)	In VIVO/In VITRO	Biological target	Tissue type	Result	Tumour Control Score	Normal-tissue Sparing Score	Therapeutic Index Score
Long-term neurocognitive benefits of FLASH radiotherapy driven by reduced reactive oxygen species	5.56E+06	5.56E+06	1.80E+00	1.00E+01	1.00E+02	1.00E+00	1.00E+01	1.80E-06	Oriatron 6e (eRT6; PMB-Alcen)	Electrons	6.00E+00	In VIVO	Mice brain	Normal Tissue	Maintenance of discrimination index (DI) (cognitive function) AND longterm neurocognitive sparing		5.00E+00	
Long-term neurocognitive benefits of FLASH radiotherapy driven by reduced reactive oxygen species	6.67E+06	6.67E+06	1.80E+00	1.20E+01	1.00E+02	1.00E+00	1.20E+01	1.80E-06	Oriatron 6e (eRT6; PMB-Alcen)	Electrons	6.00E+00	In VIVO	Mice brain	Normal Tissue	Maintenance of discrimination index (DI) (cognitive function)		4.00E+00	
Long-term neurocognitive benefits of FLASH radiotherapy driven by reduced reactive oxygen species	7.78E+06	7.78E+06	1.80E+00	1.40E+01	1.00E+02	1.00E+00	1.40E+01	1.80E-06	Oriatron 6e (eRT6; PMB-Alcen)	Electrons	6.00E+00	In VIVO	Mice brain	Normal Tissue	No maintenance of discrimination index (DI) (cognitive function)		1.00E+00	
Complete Remission of Mouse Melanoma after Temporally Fractionated Microbeam Radiotherapy	1.17E+04						4.01E+02	3.43E-02	ID17 Biomedical Beamline of the European Synchrotron Radiation Facility	Synchrotron	1.04E-01	In VIVO	Local irradiation of implanted B16–F10 melanoma cells	Tumour	50% ablated tumours, 27.5% responsive tumours, 12.5% non-responsive tumours	4.00E+00		
Feasibility of proton FLASH effect tested by zebrafish embryo irradiation	1.00E+02	5.00E+02					1.00E+01	1.00E-01	Horizontal fixed-beam beamline (UPTD)	Proton	2.24E+02	In VITRO	Wildtype zebrafish embryos	Normal tissue	Similar FLASH & CONV % survival, curvature & pericardial edema (within error bars), roughly no embryos have curvature/ edema at 4 days		5.00E+00	
Feasibility of proton FLASH effect tested by zebrafish embryo irradiation	1.00E+02	5.00E+02					1.50E+01	1.50E-01	Horizontal fixed-beam beamline (UPTD)	Proton	2.24E+02	In VITRO	Wildtype zebrafish embryos	Normal tissue	Similar FLASH & CONV % survival, curvature & pericardial edema (within error bars), less than 25% of embryos have curvature/ edema at 4 days		4.00E+00	
Feasibility of proton FLASH effect tested by zebrafish embryo irradiation	1.00E+02	5.00E+02					2.30E+01	2.30E-01	Horizontal fixed-beam beamline (UPTD)	Proton	2.24E+02	In VITRO	Wildtype zebrafish embryos	Normal tissue	Similar FLASH & CONV % survival & curvature (within error bars), FLASH less % of pericardial edema (specifically by 3/4 days post RT), less than 60% of embryos have curvature/ edema at 4 days		3.00E+00	
Feasibility of proton FLASH effect tested by zebrafish embryo irradiation	1.00E+02	5.00E+02					3.10E+01	3.10E-01	Horizontal fixed-beam beamline (UPTD)	Proton	2.24E+02	In VITRO	Wildtype zebrafish embryos	Normal tissue	Similar FLASH & CONV % survival, curvature & pericardial edema (within error bars), less than 100% of embryos have curvature/ edema at 4 days		2.00E+00	
Feasibility of proton FLASH effect tested by zebrafish embryo irradiation	1.00E+02	5.00E+02					4.20E+01	4.20E-01	Horizontal fixed-beam beamline (UPTD)	Proton	2.24E+02	In VITRO	Wildtype zebrafish embryos	Normal tissue	Similar FLASH & CONV % survival, curvature & pericardial edema (within error bars), 100% of embryos have curvature/ edema at 4 days		1.00E+00	
Effects of dose-rate on the radiation response of rat skin	6.67E+01						1.97E+01	2.95E-01		Electrons	7.00E+00	In VIVO	Hind feet rats	Normal Tissue	Small reduction in early skin reaction compared to CONV (<1 deformity score)		4.00E+00	
Effects of dose-rate on the radiation response of rat skin	6.67E+01						2.37E+01	3.55E-01		Electrons	7.00E+00	In VIVO	Hind feet rats	Normal Tissue	No reduction in early skin reaction compared to CONV (~1 deformity score)		4.00E+00	
Effects of dose-rate on the radiation response of rat skin	6.67E+01						2.75E+01	4.12E-01		Electrons	7.00E+00	In VIVO	Hind feet rats	Normal Tissue	Significant reduction in early skin reaction compared to CONV (~1 deformity score)		4.00E+00	
Effects of dose-rate on the radiation response of rat skin	6.67E+01						2.04E+01	3.05E-01		Electrons	7.00E+00	In VIVO	Hind feet rats	Normal Tissue	Significant reduction in early skin reaction, late skin reaction and deformity compared to CONV (<1.5 deformity score)		3.50E+00	
Effects of dose-rate on the radiation response of rat skin	6.67E+01						2.59E+01	3.88E-01		Electrons	7.00E+00	In VIVO	Hind feet rats	Normal Tissue	Significant reduction in early skin reaction, late skin reaction and deformity compared to CONV (~1.5 deformity score)		3.50E+00	
The constant low oxygen concentration in all the target cells for mouse tail radionecrosis	5.03E-02	3.40E+02	5.00E+00	1.70E-03	5.00E+01					Electrons	1.00E+01	In VIVO	Mouse foot skin	Normal Tissue	ND50 ~50 (almost equal to low CONV at <50)		1.00E+00	
The constant low oxygen concentration in all the target cells for mouse tail radionecrosis	7.50E-01	1.50E+04	1.00E+00	1.50E-02	5.00E+01					Electrons	1.00E+01	In VIVO	Mouse foot skin	Normal Tissue	ND50 ~55 (slightly improved compared to low CONV at <50)		2.00E+00	
The constant low oxygen concentration in all the target cells for mouse tail radionecrosis	6.64E+01	6.00E+05	5.00E-01	3.00E-01	5.00E+01					Electrons	1.00E+01	In VIVO	Mouse foot skin	Normal Tissue	ND50 ~70 (much better than CONV but still not complete sparing)		4.00E+00	
The constant low oxygen concentration in all the target cells for mouse tail radionecrosis	5.03E-02	4.00E+05	1.00E+00	4.00E-01	5.00E+01					Electrons	1.00E+01	In VIVO	Mouse foot skin	Normal Tissue	ND50 ~70 (much better than CONV but still not complete sparing)		4.00E+00	
The constant low oxygen concentration in all the target cells for mouse tail radionecrosis	2.78E+00	4.00E+03	5.00E-01	2.00E-03	5.00E+01					Electrons	1.00E+01	In VIVO	Mouse foot skin	Normal Tissue	ND50 ~ 60		3.00E+00	
The constant low oxygen concentration in all the target cells for mouse tail radionecrosis	1.49E+01	5.50E+04	1.00E+00	5.50E-02	5.00E+01					Electrons	1.00E+01	In VIVO	Mouse foot skin	Normal Tissue	ND50 ~55 (slightly improved compared to low CONV at <50)		2.00E+00	
The constant low oxygen concentration in all the target cells for mouse tail radionecrosis	1.48E+02	1.45E+05	2.00E+00	2.90E-01	5.00E+01					Electrons	1.00E+01	In VIVO	Mouse foot skin	Normal Tissue	ND50 ~70 (much better than CONV but still not complete sparing)		4.00E+00	
The constant low oxygen concentration in all the target cells for mouse tail radionecrosis	7.50E-01	6.00E+05	5.00E-01	3.00E-01	5.00E+01					Electrons	1.00E+01	In VIVO	Mouse foot skin	Normal Tissue	ND50 ~70 (much better than CONV but still not complete sparing)		4.00E+00	
The constant low oxygen concentration in all the target cells for mouse tail radionecrosis	5.03E-02	3.00E+03	5.00E+00	1.50E-02	5.00E+01					Electrons	1.00E+01	In VIVO	Mouse foot skin	Normal Tissue	ND50 ~ 60		3.00E+00	
The constant low oxygen concentration in all the target cells for mouse tail radionecrosis	6.64E+01	1.60E+03	1.00E+00	1.70E-03	5.00E+01					Electrons	1.00E+01	In VIVO	Mouse foot skin	Normal Tissue	ND50 ~50 (almost equal to low CONV at <50)		1.00E+00	
The constant low oxygen concentration in all the target cells for mouse tail radionecrosis	4.67E+01	1.00E+06	1.00E+00	1.00E+00	5.00E+01					Electrons	1.00E+01	In VIVO	Mouse foot skin	Normal Tissue	ND50 ~65		3.50E+00	
The constant low oxygen concentration in all the target cells for mouse tail radionecrosis	1.67E-01	3.50E+03	1.00E+00	3.50E-03	5.00E+01					Electrons	1.00E+01	In VIVO	Mouse foot skin	Normal Tissue	ND50 ~ 60		3.00E+00	
FLASH irradiation enhances the therapeutic index of abdominal radiotherapy for the treatment of ovarian cancer	2.16E+02	4.00E+05	5.00E+00	2.00E+00	1.08E+02	7.00E+00	1.40E+01	6.48E-02	Varian Trilogy LINAC	Electrons	1.60E+01	In VIVO	Mice total abdomen	Tumour	Tumour weight slightly more than CONV but number of tumours less (overall similar) bowel movement post FLASH worse than before irradiation but better than CONV	3.50E+00	3.50E+00	3.50E+00
FLASH irradiation enhances the therapeutic index of abdominal radiotherapy for the treatment of ovarian cancer	2.16E+02	4.00E+05	5.00E+00	2.00E+00	1.08E+02	8.00E+00	1.60E+01	7.41E-02	Varian Trilogy LINAC	Electrons	1.60E+01	In VIVO	Mice total abdomen	Normal Tissue	2x presence of regenerating crypts within the jejunum compared to CONV, Hct Hgb WBC & platelets close to control unlike CONV, still only 90% of mice recovered and survived more than 90 days post-irradiation (90% more than CONV)		3.00E+00	
FLASH irradiation enhances the therapeutic index of abdominal radiotherapy for the treatment of ovarian cancer	2.16E+02	4.00E+05	5.00E+00	2.00E+00	1.08E+02	7.00E+00	1.40E+01	6.48E-02	Varian Trilogy LINAC	Electrons	1.60E+01	In VIVO	Mice total abdomen	Normal Tissue	Epithelial integrity preserved, less severe decrement and faster recovery		4.00E+00	
[illegible]	[illegible]	[illegible]	[illegible]	[illegible]	[illegible]	[illegible]	[illegible]	[illegible]	[illegible]	[illegible]	[illegible]	[illegible]	[illegible]	[illegible]	[illegible]	[illegible]	[illegible]	[illegible]
Pencil beam scanning proton FLASH maintains tumor control while normal tissue damage is reduced in a mouse model	7.14E+01	[illegible]	[illegible]	[illegible]	[illegible]	[illegible]	[illegible]	[illegible]	[illegible]	[illegible]	[illegible]	[illegible]	[illegible]	[illegible]	[illegible]	[illegible]	[illegible]	[illegible]
Beam pulse structure and dose rate as determinants for the flash effect observed in zebrafish embryo	2.87E+02	4.40E+06	5.58E-06	2.45E-05	1.30E+07	[illegible]	[illegible]	[illegible]	[illegible]	[illegible]	[illegible]	[illegible]	[illegible]	[illegible]	[illegible]	[illegible]	[illegible]	[illegible]
Beam pulse structure and dose rate as determinants for the flash effect observed in zebrafish embryo	1.77E+02	1.40E+09	5.59E-06	7.83E-03	2.52E+04	[illegible]	[illegible]	[illegible]	[illegible]	[illegible]	[illegible]	[illegible]	[illegible]	[illegible]	[illegible]	[illegible]	[illegible]	[illegible]
Beam pulse structure and dose rate as determinants for the flash effect observed in zebrafish embryo	2.50E+05	1.50E+09	5.44E-06	8.16E-03	1.31E+07	[illegible]	[illegible]	[illegible]	[illegible]	[illegible]	[illegible]	[illegible]	[illegible]	[illegible]	[illegible]	[illegible]	[illegible]	[illegible]
Beam pulse structure and dose rate as determinants for the flash effect observed in zebrafish embryo	3.00E+02	1.50E+03	1.64E-03	2.47E-06	1.06E+08	[illegible]	[illegible]	[illegible]	[illegible]	[illegible]	[illegible]	[illegible]	[illegible]	[illegible]	[illegible]	[illegible]	[illegible]	[illegible]
FLASH irradiation induces lower levels of DNA damage ex vivo, an effect modulated by oxygen tension, dose, and dose rate	3.00E-01	3.50E+04	3.43E-01	1.20E-02	2.50E+01	[illegible]	[illegible]	[illegible]	[illegible]	[illegible]	[illegible]	[illegible]	[illegible]	[illegible]	[illegible]	[illegible]	[illegible]	[illegible]
FLASH irradiation induces lower levels of DNA damage ex vivo, an effect modulated by oxygen tension, dose, and dose rate	1.00E+00	1.20E+05	3.33E-01	4.00E-02	2.50E+01	[illegible]	[illegible]	[illegible]	[illegible]	[illegible]	[illegible]	[illegible]	[illegible]	[illegible]	[illegible]	[illegible]	[illegible]	[illegible]
FLASH irradiation induces lower levels of DNA damage ex vivo, an effect modulated by oxygen tension, dose, and dose rate	3.00E+00	3.50E+05	3.43E-01	1.20E-01	2.50E+01	[illegible]	[illegible]	[illegible]	[illegible]	[illegible]	[illegible]	[illegible]	[illegible]	[illegible]	[illegible]	[illegible]	[illegible]	[illegible]
FLASH irradiation induces lower levels of DNA damage ex vivo, an effect modulated by oxygen tension, dose, and dose rate	1.00E+01	9.80E+05	3.37E-02	3.30E-02	3.00E+02	[illegible]	[illegible]	[illegible]	[illegible]	[illegible]	[illegible]	[illegible]	[illegible]	[illegible]	[illegible]	[illegible]	[illegible]	[illegible]
FLASH irradiation induces lower levels of DNA damage ex vivo, an effect modulated by oxygen tension, dose, and dose rate	3.00E+01	2.90E+05	3.45E-01	1.00E-01	3.00E+02	[illegible]	[illegible]	[illegible]	[illegible]	[illegible]	[illegible]	[illegible]	[illegible]	[illegible]	[illegible]	[illegible]	[illegible]	[illegible]
FLASH irradiation induces lower levels of DNA damage ex vivo, an effect modulated by oxygen tension, dose, and dose rate	1.00E+02	9.60E+05	3.44E-01	3.30E-01	3.00E+02	[illegible]	[illegible]	[illegible]	[illegible]	[illegible]	[illegible]	[illegible]	[illegible]	[illegible]	[illegible]	[illegible]	[illegible]	[illegible]
FLASH irradiation induces lower levels of DNA damage ex vivo, an effect modulated by oxygen tension, dose, and dose rate	3.00E+02	2.80E+06	3.39E-01	9.50E-01	3.00E+02	[illegible]	[illegible]	[illegible]	[illegible]	[illegible]	[illegible]	[illegible]	[illegible]	[illegible]	[illegible]	[illegible]	[illegible]	[illegible]
FLASH irradiation induces lower levels of DNA damage ex vivo, an effect modulated by oxygen tension, dose, and dose rate	1.00E+03	8.40E+06	3.45E-01	2.90E+00	3.00E+02	[illegible]	[illegible]	[illegible]	[illegible]	[illegible]	[illegible]	[illegible]	[illegible]	[illegible]	[illegible]	[illegible]	[illegible]	[illegible]
FLASH irradiation induces lower levels of DNA damage ex vivo, an effect modulated by oxygen tension, dose, and dose rate	2.00E+03	1.50E+06	3.40E+00	5.00E+00	3.00E+02	[illegible]	[illegible]	[illegible]	[illegible]	[illegible]	[illegible]	[illegible]	[illegible]	[illegible]	[illegible]	[illegible]	[illegible]	[illegible]
FLASH irradiation induces lower levels of DNA damage ex vivo, an effect modulated by oxygen tension, dose, and dose rate	2.00E+03	1.50E+06	3.40E+00	5.00E+00	3.00E+02	[illegible]	[illegible]	[illegible]	[illegible]	[illegible]	[illegible]	[illegible]	[illegible]	[illegible]	[illegible]	[illegible]	[illegible]	[illegible]
FLASH irradiation induces lower levels of DNA damage ex vivo, an effect modulated by oxygen tension, dose, and dose rate	2.00E+03	1.50E+06	3.40E+00	5.00E+00	3.00E+02	[illegible]	[illegible]	[illegible]	[illegible]	[illegible]	[illegible]	[illegible]	[illegible]	[illegible]	[illegible]	[illegible]	[illegible]	[illegible]
FLASH irradiation induces lower levels of DNA damage ex vivo, an effect modulated by oxygen tension, dose, and dose rate	2.00E+03	1.50E+06	3.40E+00	5.00E+00	3.00E+02	[illegible]	[illegible]	[illegible]	[illegible]	[illegible]	[illegible]	[illegible]	[illegible]	[illegible]	[illegible]	[illegible]	[illegible]	[illegible]
FLASH irradiation induces lower levels of DNA damage ex vivo, an effect modulated by oxygen tension, dose, and dose rate	2.00E+03	1.50E+06	3.40E+00	5.00E+00	3.00E+02	[illegible]	[illegible]	[illegible]	[illegible]	[illegible]	[illegible]	[illegible]	[illegible]	[illegible]	[illegible]	[illegible]	[illegible]	[illegible]
FLASH irradiation induces lower levels of DNA damage ex vivo, an effect modulated by oxygen tension, dose, and dose rate	2.00E+03	1.50E+06	3.40E+00	5.00E+00	3.00E+02	[illegible]	[illegible]	[illegible]	[illegible]	[illegible]	[illegible]	[illegible]	[illegible]	[illegible]	[illegible]	[illegible]	[illegible]	[illegible]
Preclinical Ultra-High Dose Rate (FLASH) Proton Radiotherapy System for Small Animal Studies	6.50E+01	[illegible]	[illegible]	[illegible]	[illegible]	[illegible]	[illegible]	[illegible]	[illegible]	[illegible]	[illegible]	[illegible]	[illegible]	[illegible]	[illegible]	[illegible]	[illegible]	[illegible]
Preclinical Ultra-High Dose Rate (FLASH) Proton Radiotherapy System for Small Animal Studies	6.50E+01	[illegible]	[illegible]	[illegible]	[illegible]	[illegible]	[illegible]	[illegible]	[illegible]	[illegible]	[illegible]	[illegible]	[illegible]	[illegible]	[illegible]	[illegible]	[illegible]	[illegible]
Ultra-high dose-rate proton FLASH improves tumor control	6.00E+01	[illegible]	[illegible]	[illegible]	[illegible]	[illegible]	[illegible]	[illegible]	[illegible]	[illegible]	[illegible]	[illegible]	[illegible]	[illegible]	[illegible]	[illegible]	[illegible]	[illegible]
Antitumor Effect by Either FLASH or Conventional Dose Rate Irradiation Involves Equivalent Immune Responses		[illegible]	[illegible]	[illegible]	[illegible]	[illegible]	[illegible]	[illegible]	[illegible]	[illegible]	[illegible]	[illegible]	[illegible]	[illegible]	[illegible]	[illegible]	[illegible]	[illegible]
Antitumor Effect by Either FLASH or Conventional Dose Rate Irradiation Involves Equivalent Immune Responses		[illegible]	[illegible]	[illegible]	[illegible]	[illegible]	[illegible]	[illegible]	[illegible]	[illegible]	[illegible]	[illegible]	[illegible]	[illegible]	[illegible]	[illegible]	[illegible]	[illegible]
Antitumor Effect by Either FLASH or Conventional Dose Rate Irradiation Involves Equivalent Immune Responses		[illegible]	[illegible]	[illegible]	[illegible]	[illegible]	[illegible]	[illegible]	[illegible]	[illegible]	[illegible]	[illegible]	[illegible]	[illegible]	[illegible]	[illegible]	[illegible]	[illegible]
Proton-FLASH: effects of ultra-high dose rate irradiation on an in-vivo mouse ear model	9.30E+02	[illegible]	[illegible]	[illegible]	[illegible]	[illegible]	[illegible]	[illegible]	[illegible]	[illegible]	[illegible]	[illegible]	[illegible]	[illegible]	[illegible]	[illegible]	[illegible]	[illegible]
Proton-FLASH: effects of ultra-high dose rate irradiation on an in-vivo mouse ear model	9.30E+02	[illegible]	[illegible]	[illegible]	[illegible]	[illegible]	[illegible]	[illegible]	[illegible]	[illegible]	[illegible]	[illegible]	[illegible]	[illegible]	[illegible]	[illegible]	[illegible]	[illegible]

Key:		
	Black text	From paper
	Red text	Calculated

Table 4: % survivors of Glioma-bearing rats at 3 months post FLASH (ultra-high dose rates, dose rate of FLASH-RT recorded in columns 2 and 3) vs CONV (conventional radiotherapy, conventional dose rates). Average and standard error recorded for both modalities.

Journal Citation	Mean Dose Rate (Gy/s)	Pulse Dose Rate (Gy/s)	% survivors post FLASH	% survivors post CONV
[1]	5600000.00	5555555.56	0.00	0.00
[2]	571428.57	285714.29	50.00	48.00
[2]	595238.10	297619.05	78.80	100.00
[2]	535714.29	267857.14	100.00	93.00
[3]	66.00	133.33	100.00	85.00
[3]	74.00	73.53	0.00	0.00
		Average	54.80	54.33
		Error	7.71	7.63

Table 5: Alignment of each normal-tissue experiment with the PICO search strategy.

Ref	Population	Intervention	Comparison	Outcome
[4]	In VIVO: Mouse Brain	FLASH Radiotherapy-Electrons	Compared to before irradiation/control	Congitive function measured via Discrimination Index
[5]	In VIVO: Mouse Brain	FLASH Radiotherapy-Electrons	Compared to before irradiation/control	Cognitive function, pro-inflammatory cytokines, dendric spine density and CD68-positive microglia measurement
[6]	In VIVO: Mouse Brain	FLASH Radiotherapy-X-rays	Compared to before irradiation/control	Cognitive funtion, hippocampal cell division and indiction of reactive astrogliosis measured
[1]	In VIVO: Mouse Brain	FLASH Radiotherapy-Electrons	Compared to before irradiation/control	Cognitive function
[7]	In VIVO: Mouse Brain	FLASH Radiotherapy-Electrons	Compared to before irradiation/control	Congitive function measured via recognition ratio
[8]	In VIVO: Mouse Brain	FLASH Radiotherapy-Electrons	Compared to before irradiation/control	Microglia activation measured
[9]	In VIVO: Mouse Brain	FLASH Radiotherapy-Electrons	Compared to before irradiation/control	Astrogliosis measured
[10]	In VIVO: Mouse Brain	FLASH Radiotherapy-Electrons	Compared to before irradiation/control	LTP measured
[11]	In VIVO: Mouse Abdomen	FLASH Radiotherapy-Electrons	Compared to before irradiation/control	Regenerating crypts, intestinal mucosa, gastrointestinal function, epithelial integrity and apoptosis
[12]	In VIVO: Mouse Abdomen	FLASH Radiotherapy-Protons	Compared to before irradiation/control	Proliferating cells in intestinal crypts measured
[13]	In VIVO: Mouse Abdomen	FLASH Radiotherapy-Electrons	Compared to before irradiation/control	Survival examined
[11]	In VIVO: Mouse Abdomen	FLASH Radiotherapy-Electrons	Compared to before irradiation/control	Stool evaluated post RT
[14]	In VIVO: Mouse Abdomen	FLASH Radiotherapy-Electrons	Compared to before irradiation/control	Survival, inflammation & collagen fiber regeneration measured
[15]	In VIVO: Mouse Skin	FLASH Radiotherapy-Electrons	Compared to before irradiation/control	Desquamation measured
[16]	In VIVO: Mouse Skin	FLASH Radiotherapy-Protons	Compared to before irradiation/control	TGF-β/pro-inflammatory cytokines & desquamation/skin toxicity measured
[17]	In VIVO: Mouse Skin	FLASH Radiotherapy-Protons	Compared to before irradiation/control	Fibrosis and skin tonxicity measured
[18]	In VIVO: Mouse Skin	FLASH Radiotherapy-Electrons	Compared to control	ND50 (dose at which necrosis is observed in 50% of the animals)
[19]	In VIVO: Mouse Skin	FLASH Radiotherapy-Electrons	Compared to before irradiation/control	Depigmentation & alopecia scored
[20]	In VIVO: Mouse Skin	FLASH Radiotherapy-Protons	Compared to before irradiation	Degree of swelling measured
[21]	In VIVO: Mouse Lung	FLASH Radiotherapy-Electrons	Compared to before irradiation/control	Chromosomal damage measured
[14]	In VIVO: Mouse Lung	FLASH Radiotherapy-Electrons	Compared to before irradiation/control	Survival, inflammation & collagen fiber regeneration measured
[22]	In VIVO: Mouse Lung	FLASH Radiotherapy-Electrons	Compared to before irradiation/control	Fibrosis measured
[23]	In VIVO: Mouse Lung	FLASH Radiotherapy-Electrons	Compared to before irradiation/control	Myeloid and cytotoxic T cells measured

[12]	In VIVO: Mouse Intestine	FLASH Radiotherapy-Protons	Compared to before irradiation/control	Intestinal fibrosis measured
[24]	In VIVO: Rat Skin	FLASH Radiotherapy-Electrons	Compared to before irradiation/control	Skin toxicity measured
[25]	In VIVO: Mouse Thorax	FLASH Radiotherapy-Electrons	Compared to before irradiation/control	Fibrosis, edema and cachexia measured
[21]	In VIVO: Mouse Lung	FLASH Radiotherapy-Electrons	Compared to before irradiation/control	Fibrosis measured
[13]	In VIVO: Mouse Heart	FLASH Radiotherapy-Electrons	Compared to before irradiation/control	Lymphocyte sparing measured
[13]	In VIVO: Mouse Spleen	FLASH Radiotherapy-Electrons	Compared to before irradiation/control	Lymphocyte sparing measured
[26]	In VIVO: Cat Skin	FLASH Radiotherapy-Electrons	Compared to before irradiation/control	Erythema, moist desquamation and depilation measured
[26]	In VIVO: Minipig Skin	FLASH Radiotherapy-Electrons	Compared to before irradiation/control	Depilation, regrowth, depilation, fibrosis and necrosis measured
[2]	In VIVO: Rat Brain	FLASH Radiotherapy-Electrons	Compared to before irradiation/control	Desquamation measured
[27]	In VIVO: Mouse Blood	FLASH Radiotherapy-Electrons	Compared to before irradiation/control	Normal hematopoiesis measured
[28]	In VIVO: Mouse Pelvis	FLASH Radiotherapy-Protons	Compared to before irradiation/control	Cell proliferation measured
[29]	In VITRO: Zebrafish Embryo	FLASH Radiotherapy-Protons	Compared to before irradiation/control	Edema measured
[30]	In VITRO: Zebrafish Embryo	FLASH Radiotherapy-Protons	Compared to before irradiation/control	Edema & spinal curvature measured
[31]	In VITRO: Human IMR-90 Lung Cell Line	FLASH Radiotherapy-Protons	Compared to before irradiation/control	Clonogenic survival, γH2AX foci formation, senescence cells and pro-inflammatory marker TGFß measured
[21]	In VITRO: Human MRC5, IMR-90, A549 Lung Cell Line	FLASH Radiotherapy-Protons	Compared to before irradiation/control	Cell sparing measured
[32]	In VITRO: Human Blood	FLASH Radiotherapy-Electrons	Compared to before irradiation/control	Comet tail measured
[33]	In VITRO: Human Epithelium cell line 184A1	FLASH Radiotherapy-Electrons	Compared to before irradiation/control	Clonogenic fraction measured
[34]	In VITRO: Human HeLa cell line	FLASH Radiotherapy-Electrons	Compared to before irradiation/control	G2 cell, apoptosis and colony formation measured

Table 6: Alignment of each tumour experiment with the PICO search strategy.

Ref	Population	Intervention	Comparison	Outcome
[26]	In VIVO: Skin Tumour (T2/T3N0M0 squamous cell carcinoma) Implanted in in Cat	FLASH Radiotherapy-Electrons	Compared to before irradiation/control	Tumour recurrence measured
[2]	In VIVO: Brain Tumour (NS1 rat glioma) Implanted in Rat	FLASH Radiotherapy-Electrons	Compared to before irradiation/control	Tumour volume measured post RT
[35]	In VIVO: Brain Tumour (Glioblastoma GL261/Human U-87 MG) Implanted in Mouse	FLASH Radiotherapy-Electrons	Compared to before irradiation/control	Tumour growth measured post RT
[36]	In VIVO: Skin Tumour (B16–F10 melanoma cells) Implanted in Mouse	FLASH Radiotherapy-X-rays	Compared to before irradiation/control	Tumour volume measured post RT
[16]	In VIVO: Skin Tumour (Mouse Oral Squamous Cell Carcinoma) Implanted in Mouse	FLASH Radiotherapy-Protons	Compared to before irradiation/control	Tumour volume measured post RT
[28]	In VIVO: Skin Tumour (B-16 Flank Tumour) Implanted in Mouse	FLASH Radiotherapy-Protons	Compared to before irradiation/control	Tumour volume measured post RT
[11]	In VIVO: Ovarian Tumour (ID8) Implanted in Mouse	FLASH Radiotherapy-Electrons	Compared to before irradiation/control	Tumour weight & count measured post RT
[37]	In VIVO: Head & Neck Tumour (FaDu) Implanted in Mouse	FLASH Radiotherapy-Protons	Compared to before irradiation/control	Tumour volume measured post RT
[22]	In VIVO: Head & Neck Tumour (HEp-2 xenografts) Implanted in Mouse	FLASH Radiotherapy-Electrons	Compared to before irradiation/control	Tumour volume measured post RT
[17]	In VIVO: Breast Tumour (C3H mouse mammary carcinoma) Implanted in Mouse	FLASH Radiotherapy-Protons	Compared to before irradiation/control	Tumour recurrence measured
[14]	In VIVO: Breast Tumour (EMT6) Implanted in Mouse	FLASH Radiotherapy-Electrons	Compared to before irradiation/control	Tumour volume measured post RT
[22]	In VIVO: Breast Tumour (HBCx-12A ductal carcinoma) Implanted in Mouse	FLASH Radiotherapy-Electrons	Compared to before irradiation/control	Tumour volume measured post RT
[38]	In VIVO: Lung Tumour (LLC Lewis Lung Carcinoma) Implanted in Mouse	FLASH Radiotherapy-Protons	Compared to before irradiation/control	Tumour volume measured post RT
[22]	In VIVO: Lung Tumour (TC-1 cells (C57BL/6J mouse lung carcinoma) Implanted in Mouse	FLASH Radiotherapy-Electrons	Compared to before irradiation/control	Tumour volume measured post RT
[39]	In VIVO: Lung Tumour (LLC-mCherry tumor cells) Implanted in Mouse Lung)	FLASH Radiotherapy-Protons	Compared to before irradiation	Tumour volume measured post RT

[27]	In VIVO: Blood Cancer (Human M106 T-cells) Implanted in Mouse	FLASH Radiotherapy-Electrons	Compared to before irradiation/control	Tumuor growth and survival measured
[40]	In VITRO: Human DU145 Prostate Cancer Cells	FLASH Radiotherapy-Electrons	Compared to before irradiation/control	Surviving fraction of tumour cells
[33]	In VITRO: Human FaDu Head & Neck Cancer Cells	FLASH Radiotherapy-Electrons	Compared to before irradiation/control	Clonogenic survival & γh2AX measured
[13]	In VITRO: Mice KPC & Panc02 Pancreatic Cancer Cells	FLASH Radiotherapy-Electrons	Compared to before irradiation/control	Surviving fraction of tumour cells
[12]	In VITRO: Mice MH641905 Pancreatic Flank Cancer Cells	FLASH Radiotherapy-Protons	Compared to before irradiation/control	Tumour volume measured post RT
[41]	In VITRO: Human MCF-7 Breast Adenocarcinoma Cells	FLASH Radiotherapy-Electrons	Compared to before irradiation/control	Surviving fraction of tumour cells

References

[1] P. Montay-Gruel, M. M. Acharya, P. Gonçalves Jorge, B. Petit, I. G. Petridis, P. Fuchs, R. Leavitt, K. Petersson, M. Gondré, J. Ollivier, R. Moeckli, F. Bochud, C. Bailat, J. Bourhis, J.-F. Germond, C. L. Limoli, and M.-C. Vozenin, "Hypofractionated FLASH-RT as an effective treatment against glioblastoma that reduces neurocognitive side effects in mice," *Clin. Cancer Res.* **27** (Feb., 2021) 775–784.

[2] E. Konradsson, E. Liljedahl, E. Gustafsson, G. Adrian, S. Beyer, S. E. Ilaahi, K. Petersson, C. Ceberg, and H. Nittby Redebrandt, "Comparable long-term tumor control for hypofractionated FLASH versus conventional radiation therapy in an immunocompetent rat glioma model," *Adv. Radiat. Oncol.* **7** (Nov., 2022) 101011.

[3] E. Liljedahl, E. Konradsson, E. Gustafsson, K. F. Jonsson, J. K. Olofsson, C. Ceberg, and H. N. Redebrandt, "Long-term anti-tumor effects following both conventional radiotherapy and FLASH in fully immunocompetent animals with glioblastoma," *Sci. Rep.* **12** (July, 2022) 12285.

[4] P. Montay-Gruel, M. M. Acharya, K. Petersson, L. Alikhani, C. Yakkala, B. D. Allen, J. Ollivier, B. Petit, P. G. Jorge, A. R. Syage, T. A. Nguyen, A. A. D. Baddour, C. Lu, P. Singh, R. Moeckli, F. Bochud, J.-F. Germond, P. Froidevaux, C. Bailat, J. Bourhis, M.-C. Vozenin, and C. L. Limoli, "Long-term neurocognitive benefits of FLASH radiotherapy driven by reduced reactive oxygen species," *Proc. Natl. Acad. Sci. U. S. A.* **116** (May, 2019) 10943–10951.

[5] D. A. Simmons, F. M. Lartey, E. Schüler, M. Rafat, G. King, A. Kim, R. Ko, S. Semaan, S. Gonzalez, M. Jenkins, P. Pradhan, Z. Shih, J. Wang, R. von Eyben, E. E. Graves, P. G. Maxim, F. M. Longo, and B. W. Loo, Jr, "Reduced cognitive deficits after FLASH irradiation of whole mouse brain are associated with less hippocampal dendritic spine loss and neuroinflammation," *Radiother. Oncol.* **139** (Oct., 2019) 4–10.

[6] P. Montay-Gruel, A. Bouchet, M. Jaccard, D. Patin, R. Serduc, W. Aim, K. Petersson, B. Petit, C. Bailat, J. Bourhis, E. Bräuer-Krisch, and M.-C. Vozenin, "X-rays can trigger the FLASH effect: Ultra-high dose-rate synchrotron light source prevents normal brain injury after whole brain irradiation in mice," *Radiother. Oncol.* **129** (Dec., 2018) 582–588.

[7] P. Montay-Gruel, K. Petersson, M. Jaccard, G. Boivin, J. Germond, B. Petit, R. Doenlen, V. Favaudon, F. Bochud, C. Bailat, J. Bourhis, and M. Vozenin, "Irradiation in a flash: Unique sparing of memory in mice after whole brain irradiation with dose rates above 100 Gy/s.," *Radiotherapy and Oncology* (2017) 365–369.

[8] Y. Alaghband, S. N. Cheeks, B. D. Allen, P. Montay-Gruel, N.-L. Doan, B. Petit, P. G. Jorge, E. Giedzinski, M. M. Acharya, M.-C. Vozenin, and C. L. Limoli, "Neuroprotection of radiosensitive juvenile mice by ultra-high dose rate FLASH irradiation," *Cancers (Basel)* **12** (June, 2020) 1671.

[9] P. Montay-Gruel, M. Markarian, B. D. Allen, J. D. Baddour, E. Giedzinski, P. G. Jorge, B. Petit, C. Bailat, M.-C. Vozenin, C. Limoli, and M. M. Acharya, "Ultra-high-dose-rate FLASH irradiation limits reactive gliosis in the brain," *Radiat. Res.* **194** (Dec., 2020) 636–645.

[10] C. L. Limoli, E. A. Kramár, A. Almeida, B. Petit, V. Grilj, J. E. Baulch, P. Ballesteros-Zebadua, B. W. Loo, Jr, M. A. Wood, and M.-C. Vozenin, "The sparing effect of FLASH-RT on synaptic plasticity is maintained in mice with standard fractionation," *Radiother. Oncol.* **186** (Sept., 2023) 109767.

[11] K. Levy, S. Natarajan, J. Wang, S. Chow, J. T. Eggold, P. Loo, R. Manjappa, F. M. Lartey, E. Schüler, L. Skinner, M. Rafat, R. Ko, A. Kim, D. A. Rawi, R. von Eyben, O. Dorigo, K. M. Casey, E. E. Graves, K. Bush, A. S. Yu, A. C. Koong, P. G. Maxim, B. W. Loo, Jr, and E. B. Rankin, "FLASH irradiation enhances the therapeutic index of abdominal radiotherapy for the treatment of ovarian cancer." Dec., 2019.

[12] E. S. Diffenderfer, I. I. Verginadis, M. M. Kim, K. Shoniyozov, A. Velalopoulou, D. Goia, M. Putt, S. Hagan, S. Avery, K. Teo, W. Zou, A. Lin, S. Swisher-McClure, C. Koch, A. R. Kennedy, A. Minn, A. Maity, T. M. Busch, L. Dong, C. Koumenis, J. Metz, and K. A. Cengel, "Design, implementation, and in vivo validation of a novel proton FLASH radiation therapy system," *Int. J. Radiat. Oncol. Biol. Phys.* **106** (Feb., 2020) 440–448.

[13] B. P. Venkatesulu, A. Sharma, J. M. Pollard-Larkin, R. Sadagopan, J. Symons, S. Neri, P. K. Singh, R. Tailor, S. H. Lin, and S. Krishnan, "Ultra high dose rate (35 Gy/sec) radiation does not spare the normal tissue in cardiac and splenic models of lymphopenia and gastrointestinal syndrome," *Sci. Rep.* **9** (Nov., 2019) 17180.

[14] F. Gao, Y. Yang, H. Zhu, J. Wang, D. Xiao, Z. Zhou, T. Dai, Y. Zhang, G. Feng, J. Li, B. Lin, G. Xie, Q. Ke, K. Zhou, P. Li, X. Shen, H. Wang, L. Yan, C. Lao, L. Shan, M. Li, Y. Lu, M. Chen, S. Feng, J. Zhao, D. Wu, and X. Du, "First demonstration of the FLASH effect with ultrahigh dose rate high-energy X-rays," *Radiother. Oncol.* **166** (Jan., 2022) 44–50.

[15] T. Inada, H. Nishio, S. Amino, K. Abe, and K. Saito, "High dose-rate dependence of early skin reaction in mouse," *Int. J. Radiat. Biol. Relat. Stud. Phys. Chem. Med.* **38** (Aug., 1980) 139–145.

[16] S. Cunningham, S. McCauley, K. Vairamani, J. Speth, S. Girdhani, E. Abel, R. A. Sharma, J. P. Perentesis, S. I. Wells, A. Mascia, and M. Sertorio, "FLASH proton pencil beam scanning irradiation minimizes radiation-induced leg contracture and skin toxicity in mice," *Cancers (Basel)* **13** (Mar., 2021).

[17] B. S. Sørensen, M. K. Sitarz, C. Ankjærgaard, J. G. Johansen, C. E. Andersen, E. Kanouta, C. Grau, and P. Poulsen, "Pencil beam scanning proton FLASH maintains tumor control while normal tissue damage is reduced in a mouse model," *Radiother. Oncol.* **175** (Oct., 2022) 178–184.

[18] J. Hendry, J. Moore, B. Hodgson, and J. Keene, "The Constant Low Oxygen Concentration in All the Target Cells for Mouse Tail Radionecrosis,".

[19] L. A. Soto, K. M. Casey, J. Wang, A. Blaney, R. Manjappa, D. Breitkreutz, L. Skinner, S. Dutt, R. B. Ko, K. Bush, A. S. Yu, S. Melemenidis, S. Strober, E. Englemann, P. G. Maxim, E. E. Graves, and B. W. Loo, "FLASH irradiation results in reduced severe skin toxicity compared to conventional-dose-rate irradiation," *Radiat. Res.* **194** (Dec., 2020) 618–624.

[20] S. Rudigkeit, T. E. Schmid, A. C. Dombrowsky, J. Stolz, S. Bartzsch, C.-B. Chen, N. Matejka, M. Sammer, A. Bergmaier, G. Dollinger, and J. Reindl, "Proton-FLASH: effects of ultra-high dose rate irradiation on an in-vivo mouse ear model," *Sci. Rep.* **14** (Jan., 2024).

[21] C. Fouillade, S. Curras-Alonso, L. Giuranno, E. Quelennec, S. Heinrich, S. Bonnet-Boissinot, A. Beddok, S. Leboucher, H. U. Karakurt, M. Bohec, S. Baulande, M. Vooijs, P. Verrelle, M. Dutreix, A. Londoño-Vallejo, and V. Favaudon, "FLASH irradiation spares lung progenitor cells and limits the incidence of radio-induced senescence," *Clin. Cancer Res.* **26** (Mar., 2020) 1497–1506.

[22] V. Favaudon, L. Caplier, V. Monceau, F. Pouzoulet, M. Sayarath, C. Fouillade, M.-F. Poupon, I. Brito, P. Hupé, J. Bourhis, J. Hall, J.-J. Fontaine, and M.-C. Vozenin, "Ultrahigh dose-rate FLASH irradiation increases the differential response between normal and tumor tissue in mice," *Sci. Transl. Med.* **6** (July, 2014) 245ra93.

[23] Y.-E. Kim, S.-H. Gwak, B.-J. Hong, J.-M. Oh, H.-S. Choi, M. S. Kim, D. Oh, F. M. Lartey, M. Rafat, E. Schüler, H.-S. Kim, R. von Eyben, I. L. Weissman, C. J. Koch, P. G. Maxim, B. W. Loo, Jr, and G.-O. Ahn, "Effects of ultra-high doserate FLASH irradiation on the tumor microenvironment in Lewis lung carcinoma: Role of myosin light chain," *Int. J. Radiat. Oncol. Biol. Phys.* **109** (Apr., 2021) 1440–1453.

[24] S. Field and D. Bewley, "Effects of Dose-rate on the Radiation Response of Rat Skin," *International Journal of Radiation Biology and Related Studies in Physics, Chemistry and Medicine* (1974) 259–267.

[25] V. Favaudon, L. Caplier, V. Monceau, F. Pouzoulet, M. Sayarath, C. Fouillade, M.-F. Poupon, I. Brito, P. Hupé, J. Bourhis, J. Hall, J.-J. Fontaine, and M.-C. Vozenin, "Ultrahigh dose-rate FLASH irradiation increases the differential response between normal and tumor tissue in mice," *Sci. Transl. Med.* **6** (July, 2014) 245ra93.

[26] M. Vozenin, P. De Fornel, K. Petersson, V. Favaudon, M. Jaccard, J. Germond, B. Petit, M. Burki, G. Ferrand, D. Patin, H. Bouchaab, M. Ozsahin, F. Bochud, C. Bailat, P. Devauchelle, and J. Bourhis, "The Advantage of FLASH Radiotherapy Confirmed in Mini-pig and Cat-cancer Patients.,".

[27] S. Chabi, T. H. Van To, R. Leavitt, S. Poglio, P. G. Jorge, M. Jaccard, K. Petersson, B. Petit, P.-H. Roméo, F. Pflumio, M.-C. Vozenin, and B. Uzan, "Ultra-high-dose-rate FLASH and conventional-dose-rate irradiation differentially affect human acute lymphoblastic leukemia and normal hematopoiesis," *Int. J. Radiat. Oncol. Biol. Phys.* **109** (Mar., 2021) 819–829.

[28] N. Cao, D. P. J. Erickson, E. C. Ford, R. C. Emery, M. Kranz, P. Goff, M. Schwarz, J. Meyer, T. Wong, J. Saini, C. Bloch, R. D. Stewart, G. A. Sandison, A. Morimoto, A. DeLonais-Dick, B. A. Shaver, R. Rengan, and J. Zeng, "Preclinical ultra-high dose rate (FLASH) proton radiotherapy system for small animal studies," *Adv. Radiat. Oncol.* (Dec., 2023) 101425.

[29] E. Beyreuther, M. Brand, S. Hans, K. Hideghéty, L. Karsch, E. Leßmann, M. Schürer, E. R. Szabó, and J. Pawelke, “Feasibility of proton FLASH effect tested by zebrafish embryo irradiation,” *Radiother. Oncol.* **139** (Oct., 2019) 46–50.

[30] L. Karsch, J. Pawelke, M. Brand, S. Hans, K. Hideghéty, J. Jansen, E. Lessmann, S. Löck, M. Schürer, R. Schurig, J. Seco, E. R. Szabó, and E. Beyreuther, “Beam pulse structure and dose rate as determinants for the flash effect observed in zebrafish embryo,” *Radiother. Oncol.* **173** (Aug., 2022) 49–54.

[31] M. Buonanno, V. Grilj, and D. J. Brenner, “Biological effects in normal cells exposed to FLASH dose rate protons,” *Radiother. Oncol.* **139** (Oct., 2019) 51–55.

[32] C. R. Cooper, D. Jones, G. D. Jones, and K. Petersson, “FLASH irradiation induces lower levels of DNA damage ex vivo, an effect modulated by oxygen tension, dose, and dose rate,” *Br. J. Radiol.* **95** (May, 2022) 20211150.

[33] L. Laschinsky, M. Baumann, E. Beyreuther, W. Enghardt, M. Kaluza, L. Karsch, E. Lessmann, D. Naumburger, M. Nicolai, C. Richter, R. Sauerbrey, H.-P. Schlenvoigt, and J. Pawelke, “Radiobiological effectiveness of laser accelerated electrons in comparison to electron beams from a conventional linear accelerator,” *J. Radiat. Res.* **53** (May, 2012) 395–403.

[34] S. Auer, V. Hable, C. Greubel, G. A. Drexler, T. E. Schmid, C. Belka, G. Dollinger, and A. A. Friedl, “Survival of tumor cells after proton irradiation with ultra-high dose rates,” *Radiat. Oncol.* **6** (Oct., 2011) 139.

[35] A. Almeida, C. Godfroid, R. J. Leavitt, P. Montay-Gruel, B. Petit, J. Romero, J. Ollivier, L. Meziani, K. Sprengers, R. Paisley, V. Grilj, C. L. Limoli, P. Romero, and M.-C. Vozenin, “Anti-tumor effect by either FLASH or conventional dose rate irradiation involves equivalent immune responses,” *Int. J. Radiat. Oncol. Biol. Phys.* (Nov., 2023).

[36] C. Fernandez-Palomo, V. Trappetti, M. Potez, P. Pellicioli, M. Krisch, J. Laissue, and V. Djonov, “Complete remission of mouse melanoma after temporally fractionated Microbeam Radiotherapy,” *Cancers (Basel)* **12** (Sept., 2020) 2656.

[37] O. Zlobinskaya, C. Siebenwirth, C. Greubel, V. Hable, R. Hertenberger, N. Humble, S. Reinhardt, D. Michalski, B. Röper, G. Multhoff, G. Dollinger, J. J. Wilkens, and T. E. Schmid, “The effects of ultra-high dose rate proton irradiation on growth delay in the treatment of human tumor xenografts in nude mice,” *Radiat. Res.* **181** (Feb., 2014) 177–183.

[38] N. Rama, T. Saha, S. S., G. C., M. D., M. A., V. R., S. D., M. A. Katsis, E. Abel, S. Girdhani, M. M., A. Rodriguez, A. Ku, R. Dua, R. Parry, and T. Kalin, “Improved Tumor Control Through T-cell Infiltration Modulated by Ultra-High Dose Rate Proton FLASH Using a Clinical Pencil Beam Scanning Proton System.,” *International Journal of Radiation Oncology*Biology*Physics* (2019) 164–165.

[39] S. Shukla, T. Saha, N. Rama, A. Acharya, T. Le, F. Bian, J. Donovan, L. A. Tan, R. Vatner, V. Kalinichenko, A. Mascia, J. P. Perentesis, and T. V. Kalin, “Ultra-high dose-rate proton FLASH improves tumor control,” *Radiother. Oncol.* **186** (Sept., 2023) 109741.

[40] G. Adrian, E. Konradsson, M. Lempart, S. Bäck, C. Ceberg, and K. Petersson, “The FLASH effect depends on oxygen concentration,” *Br. J. Radiol.* **93** (Feb., 2020) 20190702.

[41] M. Sumini, A. Previti, D. Galassi, E. Ceccolini, F. Rocchi, D. Mostacci, A. Tartari, F. Pasi, A. Facoetti, G. Mazzini, R. Nano, A. Virelli, I. Zironi, G. Castellani, G. Cucchi, and R. Orecchia, “Analysis and characterization of the X-ray beam produced by a PF device for radiotherapy applications,” *Xray Spectrom.* **44** (July, 2015) 289–295.
